# Casein kinase 1α is required to maintain murine hypothalamic pro-opiomelanocortin expression

**DOI:** 10.1016/j.isci.2023.106670

**Published:** 2023-04-14

**Authors:** Chenyang Lu, Jinglin Zhang, Bingjie Wang, Qiao Gao, Kezhe Ma, Shaona Pei, Juxue Li, Sheng Cui

**Affiliations:** 1 College of Veterinary Medicine, Yangzhou University, Yangzhou, Jiangsu 225009, People’s Republic of China; 2 Institute of Reproduction and Metabolism, Yangzhou University, Yangzhou, Jiangsu 225009, People’s Republic of China; 3 Joint International Research Laboratory of Agriculture and Agri-Product Safety, the Ministry of Education of China, Yangzhou University, Yangzhou, Jiangsu 225009, People’s Republic of China; 4 Jiangsu Co-innovation Center for Prevention and Control of Important Animal Infectious Diseases and Zoonoses, Yangzhou, Jiangsu 225009, People’s Republic of China; 5 State Key Laboratory of Reproductive Medicine, Nanjing Medical University, Nanjing, Jiangsu 211166, China

**Keywords:** Neuroscience, Developmental neuroscience, Cellular neuroscience

## Abstract

Hypothalamic pro-opiomelanocortin (POMC) neuron development is considered to play an essential role in the development of obesity. However, the underlying mechanisms remain unclear. Casein kinase 1α (CK1α) was expressed in the embryonic mouse hypothalamus at high levels and colocalized with POMC neurons. CK1α deletion in POMC neurons caused weight gain, metabolic defects, and increased food intake. The number of POMC-expressing cells was considerably decreased in Csnk1a1^fl/fl;^POMC^cre^ (PKO) mice from embryonic day 15.5 to postnatal day 60, while apoptosis of POMC neurons was not affected. Furthermore, unchanged POMC progenitor cells and a decreased POMC phenotype established CK1α function in hypothalamic POMC neuron development. CK1α deletion led to elevated Notch intracellular domain (NICD) protein expression, and NICD inhibition rescued the PKO mouse phenotype. In summary, CK1α is involved in hypothalamic POMC expression via NICD-POMC signaling, deepening our understanding of POMC neuron development and control of systemic metabolic functions.

## Highlights

• CK1α is expressed in the embryonic mouse hypothalamus at high levels
• CK1α deletion in POMC neurons causes weight gain and metabolic defects
• CK1α is involved in hypothalamic POMC expression via NICD-POMC signaling
• Our data confirm a regulatory mechanism of POMC expression during embryogenesis

## Graphical abstract

**Figure:**
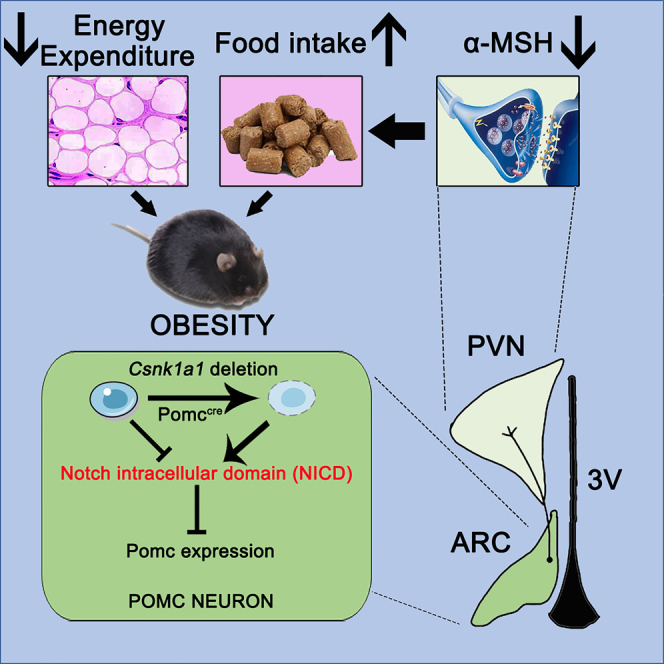

## Introduction

Hypothalamic melanocortin system development is involved in regulating energy metabolism. In the melanocortin system, POMC neurons develop through two stages: proliferation, migration, and differentiation at the embryonic stage, followed by axonal growth and synaptogenesis after birth.^1^^,^^2^^,^^3^ POMC neurons can be recognized in the anterior basal region at embryonic day (E) 10.5, and the *Pomc* mRNA expression level reaches its peek at E13.^4^ Pomc progenitors further differentiate into at least three mature neural subtypes: POMC, NPY,^5^ and kisspeptin.^6^ After birth, axonal growth of POMC occurs under the regulation of leptin^7^ or BDNF.^8^ POMC neurons in the hypothalamic arcuate nucleus (ARC) release α-melanocyte-stimulating hormone (α-MSH) to paraventricular hypothalamic nucleus (PVN) via melanocortin 4 receptors (MC4R).^9^ In the hypothalamic ARC melanocortin system, POMC,^10^ which suppresses appetite and increases energy expenditure, and neuropeptide Y/agouti-related neuropeptide (NPY/AgRP),^11^ which enhances appetite and reduces energy expenditure, work together to regulate energy balance in animals.

POMC neuron development is regulated by several factors, including Notch,^12^ Shh,^12^ Nkx2-1,^13^ and Rax^14^ during embryonic development. Notch signaling is evolutionarily conserved and regulates POMC expression and neurogenesis through intercellular communication in the embryonic development stage.^12^ When Notch receptors in neurons interact with Notch ligands (delta or serrate/jagged) in neighboring cells, the Notch intracellular domain (NICD) is released.^15^ Recombinant signal-binding protein for the immunoglobulin kappa J region (RBPJ) forms a nuclear complex with NICD, which activates the transcription of the target gene *Hes1*,^16^ thereby preventing the onset of POMC expression.^17^ Activation of NICD leads to an increasing hypothalamic progenitor cell count and decreasing differentiated POMC neurons, resulting in the decrease of POMC expression.^17^ In contrast, conditional deletion of the Notch cofactor Rbpjk in NKX2-1 cells causes a decrease in the progenitor cell count and an increase in the differentiated POMC cell count, resulting in the increase of POMC expression.^17^

CK1α, encoded by *Csnk1a1*, comes from the serine/threonine-protein kinase family.^18^^,^^19^^,^^20^ CK1α regulates multiple signaling pathways, including the circadian rhythm,^21^ autophagy,^22^^,^^23^ apoptosis,^24^^,^^25^ and developmental differentiation.^26^ CK1α functions in the maintenance of epidermal progenitors^27^ as well as in spermatogonia-committed progenitors.^28^ However, the function of CK1α in neural progenitor cells is unknown, although its expression has been reported in the hypothalamus.^26^ Therefore, we hypothesized that CK1α may control POMC neuron function and may have important implications for systemic energy metabolism. To test this hypothesis, CK1α function in murine POMC neurons was assessed and whether the loss of CK1α expression affects POMC neuron development was investigated.

Here, we report that CK1α regulates POMC expression via NICD-POMC, which is essential for the regulation of POMC neuron development.

## Results

### CK1α and pro-opiomelanocortin are co-expressed in developing and adult arcuate nucleus

**Figure 1:**
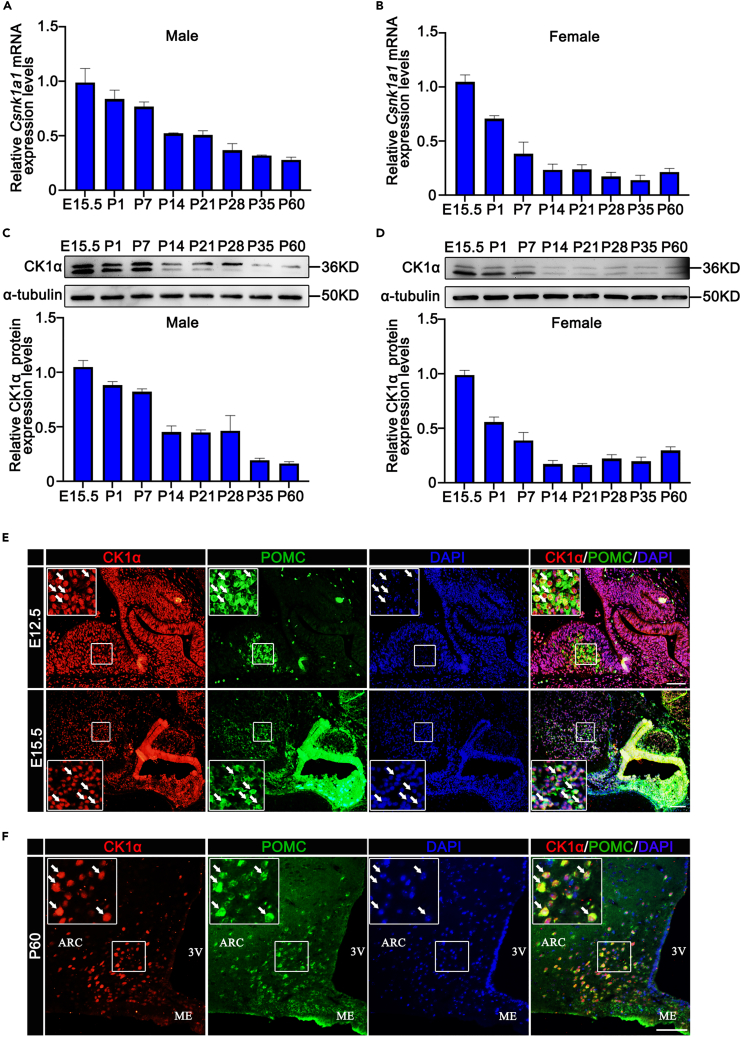
CK1α is localized in hypothalamic POMC neurons (A and B) Real-time PCR quantification of *Csnk1a1* mRNA expression levels in male and female mouse hypothalami from E15.5 to P60 (n = 3). Data are means ± SEM. (C and D) Western blotting analysis of CK1α protein expression levels in male and female mouse hypothalami from E15.5 to P60 (n = 3). Data are means ± SEM. (E and F) Immunofluorescence detection of CK1α (red) and POMC (green) in E12.5 (E), E15.5 (E), and P60 (F) mouse hypothalamus sections. Arrows indicate representative CK1α^+^ POMC^+^ cells. Scale bars, 50 μm.

To examine whether CK1α plays a role in hypothalamic development, *Csnk1a1* mRNA and CK1α protein expressions of the mouse hypothalamus were measured using real-time PCR and western blotting, respectively. In the hypothalamus of E15.5 male and female mice, we found that *Csnk1a1* mRNA and CK1α protein expression levels were at their highest values (Figures 1A-1D), suggesting that CK1α is involved in the development of embryonic hypothalami. *Csnk1a1* mRNA and CK1α protein levels then decreased from E15.5 to postnatal day (P) 60 (Figures 1A–1D). We next evaluated CK1α expression in the hypothalamus by immunofluorescence. CK1α and POMC were co-expressed in ARC as early as at E12.5 (93.4%) and E15.5 (95.6%) (Figure 1E) and continued to be expressed in adulthood (96.3%; Figures 1F and S1B, Table S1), suggesting that CK1α may affect POMC expression in mouse hypothalamic development.

### *Csnk1a1* deletion causes obesity

**Figure 2:**
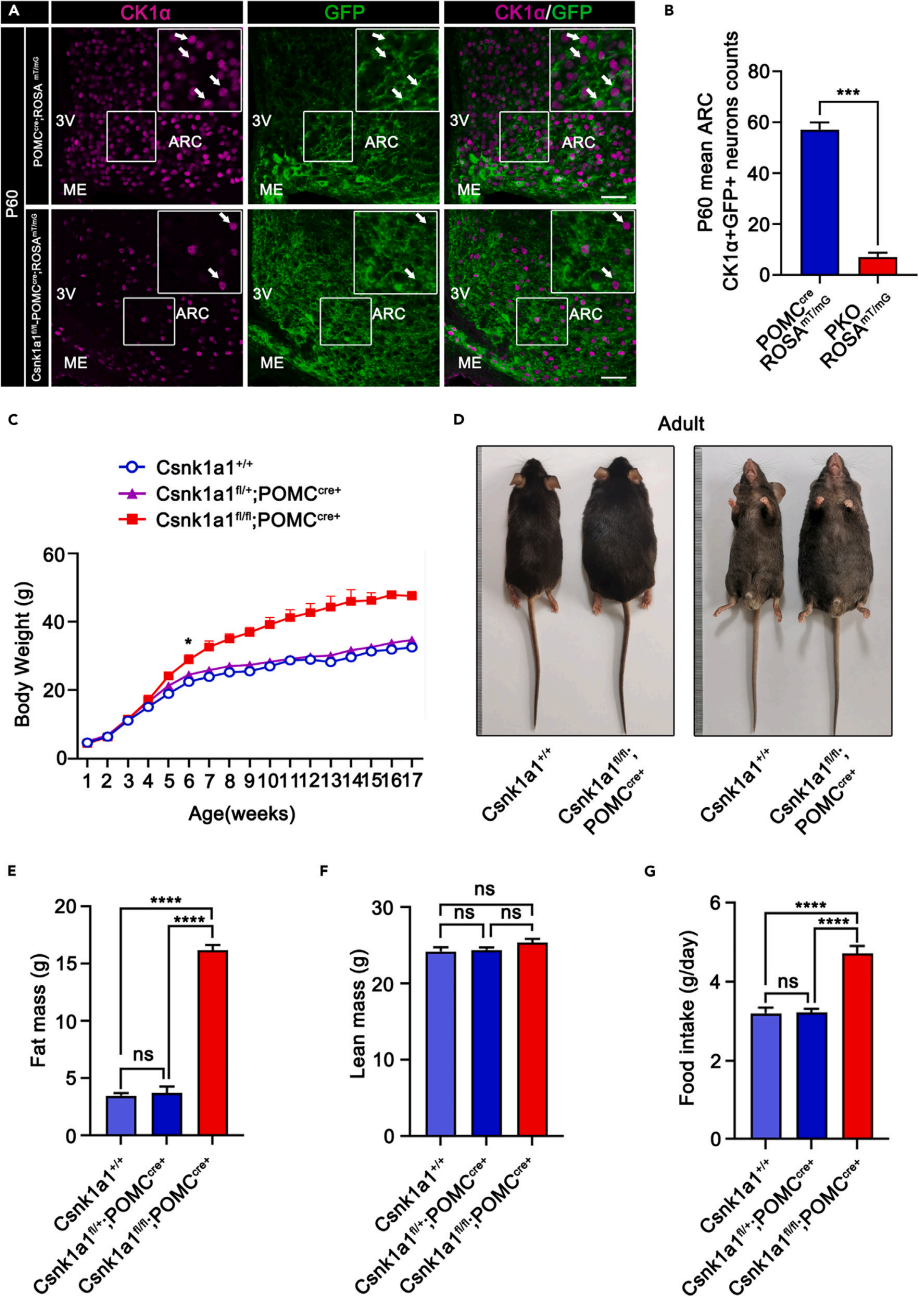
CK1α deletion in POMC neurons causes obesity (A) Immunofluorescence detection of CK1α (purple) and GFP (green) in P60 hypothalami of POMC^cre^;ROSA^mT/mG^ mice and Csnk1a1^fl/fl^;POMC^cre^;ROSA^mT/mG^ mice. Arrows indicate representative CK1α^+^GFP^+^ positive neurons. Scale bars, 50 μm. (B) Relative quantification of CK1α^+^GFP^+^ neurons (arrows) in P60 hypothalami of POMC^cre^;ROSA^mT/mG^ mice and Csnk1a1^fl/fl^;POMC^cre^;ROSA^mT/mG^ mice ARCs (n = 3, ∗∗∗p < 0.001). Data are means ± SEM. (C) Growth (body weight) of Csnk1a1^+/+^, Csnk1a1^fl/+^;POMC^cre^ and PKO mice.(n = 5, ∗p < 0.05). Data are means ± SEM. (D) Representative photographs of Csnk1a1^+/+^ and PKO mice. (E and F) Fat (E) and lean (F) masses of 12-week-old Csnk1a1^+/+^, Csnk1a1^fl/+^;POMC^cre^ and PKO mice (n = 5, ns p > 0.05, ∗∗∗∗p < 0.0001). Data are means ± SEM. (G) Daily food consumption of 12-week-old Csnk1a1^+/+^, Csnk1a1^fl/+^;POMC^cre^ and PKO mice (n = 5, ns p > 0.05, ∗∗∗∗p < 0.0001). Data are means ± SEM.

Pomc-specific CK1α knockout mice were generated by crossing *Csnk1a1*^*Flox/Flox*^ mice with *POMC*^*cre*^ mice. To evaluate deletion efficacy, ROSA^mT/mG^ reporter mice were crossed with *POMC*^*cre*^ and PKO (Csnk1a1^fl/fl^;POMC^cre^) mice for cell lineage tracing, to label cre-mediated neuronal protrusions and recombination cells permanently and genetically, and CK1α immunofluorescence staining was performed. CK1α-positive and GFP-positive cell numbers significantly decreased by 87.7% in the ARC of adult Csnk1a1^fl/fl^;POMC^cre^;ROSA^mT/mG^ mice relative to POMC^cre^;ROSA^mT/mG^ mice (Figures 2A and 2B), although CK1α was still expressed in some remaining POMC neurons in PKO mice (Figures S2A and S2B). These data indicate that *POMC*^*cre*^ deleted CK1α in POMC neurons successfully.

As *POMC*^*cre*^ mice could also recombine in the pituitary, pituitary function was examined. The pituitary gene *Pomc*, thyroid-stimulating hormone beta-chain (*Tshb*), and growth hormone (*Gh*) mRNA expressions were unchanged (Figure S3A). Consistently, immunofluorescence showed that the number of POMC-, GH-, and TSHB-positive cells were unaltered in control and PKO mouse pituitaries (Figure S3B). These results indicate that CK1α deletion did not cause pituitary defects.

To investigate whether CK1α deletion influences energy balance, we monitored the weights of Csnk1a1^+/+^, Csnk1a1^fl/+^;POMC^cre^, and PKO mice under a chow diet (CD). PKO mice were heavier starting at 6 weeks of age (Figures 2C and 2D) and had higher fat mass, whereas their lean mass component was unchanged (Figures 2E and 2F), as seen by an increase in epididymal white adipose tissue (eWAT), inguinal adipose tissue (iWAT), and brown adipose tissue (BAT; Figures S4A–S4D). The higher body weight of PKO mice was also associated with increased food intake and decreased energy expenditure (Figures 2G, S5A, and S5B). Female PKO mice exhibited similar phenotypes; therefore, we performed subsequent studies on male mice.

Obesity is associated with changes in glucose metabolism, leptin resistance, and hepatic steatosis. The 12-week-old PKO mice exhibited impaired glucose and insulin tolerance (Figures S6A–S6D). Furthermore, serum leptin levels were higher in PKO mice than in controls (Figure S7A). To examine whether *Csnk1a1* knockout affected leptin sensitivity, we injected mice with 1.5 mg/kg leptin intraperitoneally^29^ twice a day for three days and then measured the change in body weight gain and food intake. Leptin had little impact on body weight gain (Figure S7B) and cumulative food intake in PKO mice (Figure S7C) relative to that in controls. Consistently, western blotting results showed that the increase in phosphorylated signal transducer and activator of transcription 3 (p-STAT3, Tyr705) protein levels, which represents leptin signaling activity, was significantly reduced in PKO mice (Figures S7D and S7E). The expressions of leptin receptor (Lepr) mRNA and protein also decreased by 42 and 48% in PKO mice hypothalami compared with the controls (Figures S7F–S7H). In addition, the increase in liver weight, Oil Red O, and H&E staining demonstrated that CK1α knockout resulted in hepatic steatosis (Figures S8A–S8C). Together, these results demonstrate a specific role of CK1α in the regulation of energy homeostasis in POMC neurons.

### CK1α loss in pro-opiomelanocortin neurons leads to a significant decrease in the numbers of pro-opiomelanocortin-positive cells, resulting in reduced α-melanocyte-stimulating hormone expression

**Figure 3:**
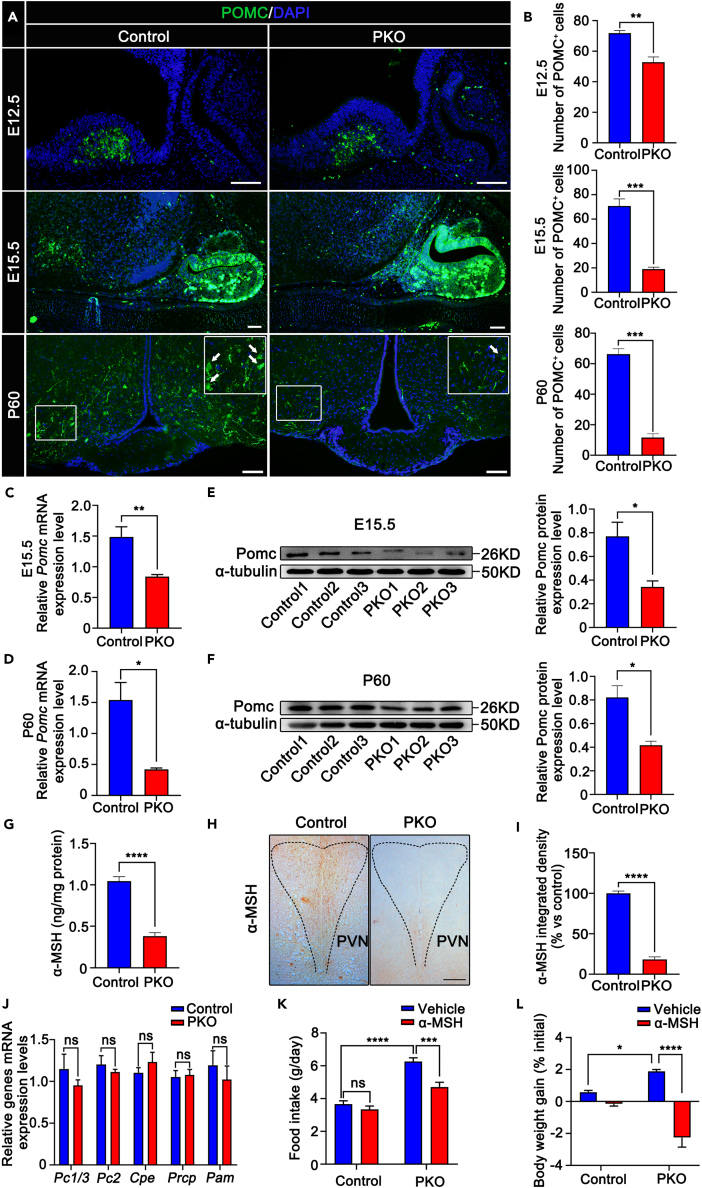
CK1α deletion reduces the number of POMC-positive cells (A and B) Immunofluorescence micrographs (A) and quantification (B) of relative numbers of POMC-expressing neurons (arrows) in control and PKO ARCs at E12.5, E15.5, and P60. Scale bars, 50 μm (n = 3, ∗∗p < 0.01, ∗∗∗p < 0.001). Data are means ± SEM. (C and D) Real-time PCR quantification of *Pomc* mRNA in control and PKO hypothalami at E15.5 (C) and P60 (D) (n = 5, ∗p < 0.05, ∗∗p < 0.01). Data are means ± SEM. (E and F) Western blotting analysis and quantification of POMC protein expression in control and PKO hypothalami at E15.5 (E) and P60 (F) (n = 3, ∗p < 0.05). Data are means ± SEM. (G) Hypothalamic α-MSH content in 12-week-old control and PKO mice (n = 5, ∗∗∗∗p < 0.0001). Data are means ± SEM. (H and I) Representative immunohistochemistry micrographs of α-MSH staining (H) in PVNs of 12-week-old mice and integrated density quantification (I) (n = 3, ∗∗∗∗p < 0.0001). Data are means ± SEM. Scale bars, 50 μm. (J) Real-time PCR quantification of POMC processing genes mRNA expressions in hypothalami of 12-week-old male control and PKO mice (n = 5, ns p > 0.05). Data are means ± SEM. (K and L) Changes in food intake (K) and body weight gain (L) after the injection of α-MSH in 12-week-old mice (n = 6, ns p > 0.05, ∗p < 0.05, ∗∗∗p < 0.001, ∗∗∗∗p < 0.0001). Data are means ± SEM. Control = Csnk1a1^fl/+^;POMC^cre^. PKO = Csnk1a1^fl/fl^;POMC^cre^.

To investigate the mechanisms underlying metabolic dysregulation in PKO mice, we examined whether the absence of CK1α in POMC neurons leads to altered neurodevelopment. Immunofluorescence was performed to count POMC^+^ neurons in the PKO ARC, which at E12.5, E15.5, and P60 were 73.5, 26.9, and 17.6% of those observed in controls, respectively (Figures 3A and 3B, and Table S2). The reduction was accompanied by reduced expressions of *Pomc* mRNA and POMC protein at E15.5 (Figures 3C and 3E) and P60 (Figures 3D and 3F) relative to those in controls, while *Agrp*, *Npy*, and *Cart* mRNA expressions were unchanged (Figures S9A and S9B).

We next examined the expression of genes encoding hypothalamic hormones. We did not observe significant changes in melanin-concentrating hormone (*Mch*), thyrotropin-releasing hormone (*Trh*), corticotropin-releasing hormone (*Crh*), or orexin (*Hcrt*) mRNA expression (Figure S9C). POMC generates α-MSH to PVN through processing enzymes to regulate energy homeostasis. Thus, we examined α-MSH peptide expression in the PVN and α-MSH content of hypothalamic extracts using immunohistochemistry and ELISA from 12-week-old control and PKO mice. The α-MSH content decreased by 63.5% (Figure 3G), and α-MSH staining decreased significantly in PKO mice neuronal projections (Figures 3H and 3I). However, the decrease in α-MSH did not result from processing enzyme expression changes, as carboxypeptidase E (*Cpe*), α-amidating monooxygenase (*Pam*), prolylcarboxypeptidase *(Prcp),* prohormone convertase 2 (*Pc2*), and prohormone convertase 1 (*Pc1/3*) mRNA levels were unaltered (Figure 3J).

To investigate whether the decrease in the α-MSH content led to obesity, recombinant α-MSH was used to improve PKO mice obesity. Daily α-MSH injections markedly reduced weight gain and food intake in PKO mice (Figures 3K and 3L). Together, the results indicate that CK1α conditional deletion results in the marked reduction of POMC^+^ neurons, and that the decrease in these α-MSH content causes the obese PKO mice phenotype.

### CK1α is essential for pro-opiomelanocortin expression in mouse arcuate nucleus

**Figure 4:**
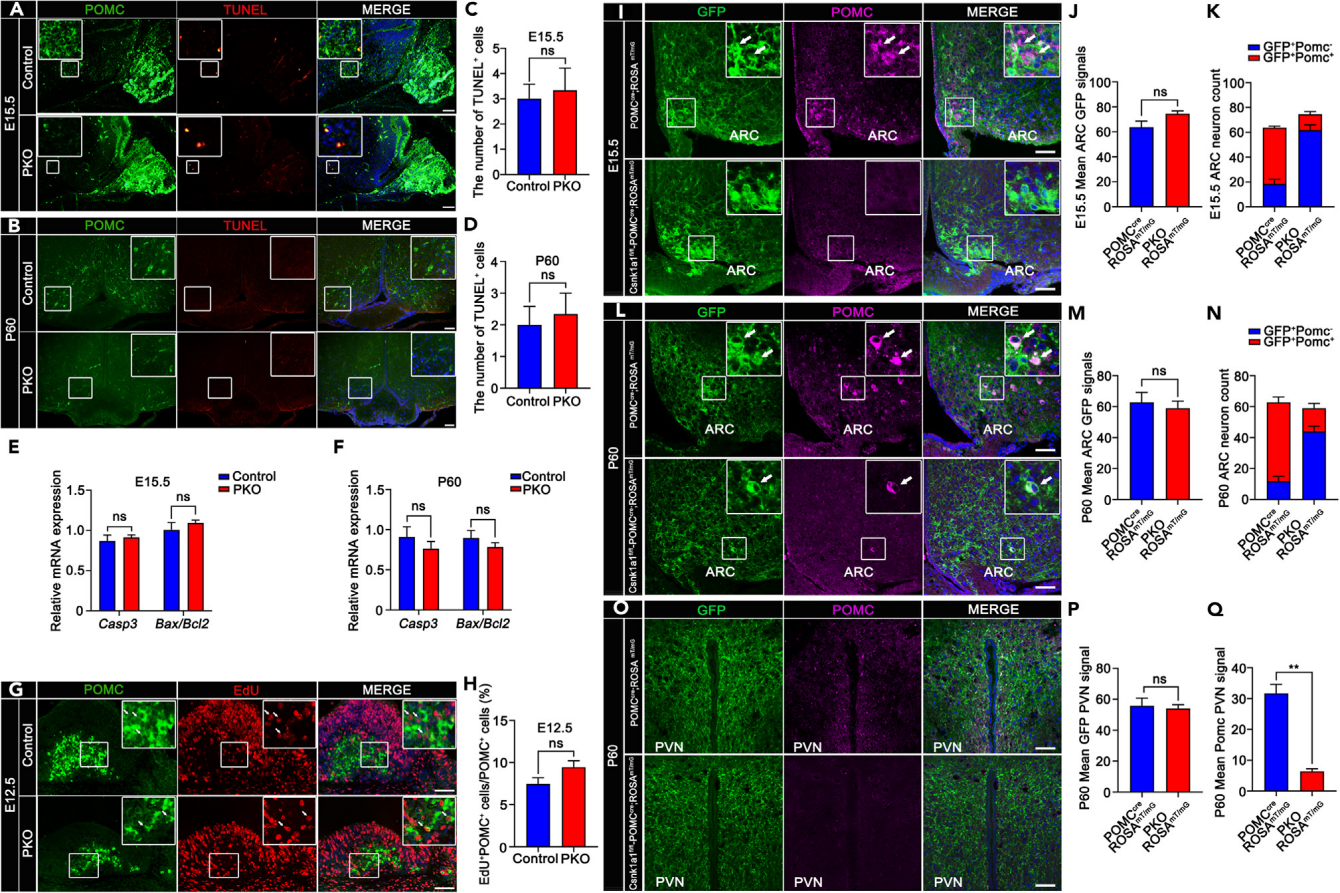
CK1α is essential to maintain POMC expression (A and B) Double immunofluorescence staining for POMC and TUNEL in control and PKO hypothalami at E15.5 (A) and P60 (B). Scale bars, 50 μm. (C and D) Quantification of relative TUNEL signals in the ARCs of control and PKO mice at E15.5 (C) and P60 (D) (n = 3, ns p > 0.05). Data are means ± SEM. (E and F) Real-time PCR quantification of apoptosis-related genes *Casp3* and *Bax/Bcl2* expression levels in 12-week-old mice hypothalami (n = 5, ns p > 0.05). Data are means ± SEM. (G) Double immunofluorescence staining for POMC and EdU in control and PKO ARC cells at E12.5. Arrows indicate representative POMC^+^ EdU^+^ neurons. Scale bars, 50 μm. (H) Ratios of POMC^+^ EdU^+^ neurons to POMC^+^ neurons in control and PKO ARCs at E12.5 (n = 3; in %; ns p > 0.05). Data are means ± SEM. (I-N) Representative micrographs and relative quantification of GFP neurons (arrows) of POMC^cre^;ROSA^mT/mG^ mice and Csnk1a1^fl/fl^;POMC^cre^;ROSA^mT/mG^ ARCs at E15.5 (I,J,K) and P60 (L,M,N) (n = 3, ns p > 0.05). Data are means ± SEM. Scale bars, 50 μm. (O-Q) Representative micrographs and relative quantification of depicting Cre recombination and Pomc-positive neuronal fibers in the adult PVN (n = 3, ns p > 0.05, ∗∗p < 0.01). Data are means ± SEM. Control = Csnk1a1^fl/+^;POMC^cre^. PKO = Csnk1a1^fl/fl^;POMC^cre^. Scale bars, 50 μm.

As previously reported in multiple neuronal systems,^30^^,^^31^ cell death or proliferation may lead to a decrease in POMC^+^ neurons during embryonic development. To test this hypothesis, we assessed apoptosis using a terminal deoxynucleotidyl transferase dUTP nick end labeling (TUNEL) assay. Compared with those of controls, no significant differences were examined in TUNEL^+^POMC^+^ neuron numbers in PKO hypothalamus ARCs at E15.5 (Figures 4A and 4C) and P60 (Figures 4B and 4D). Furthermore, we analyzed the expression of the apoptosis-related genes *Casp3* and *Bax/Bcl2* in the hypothalami of control and PKO mice. We observed no significant differences at E15.5 (Figure 4E) and P60 (Figure 4F). Consistently, western blotting results showed no difference in c-Caspase3 expression between control and PKO hypothalami at E15.5 (Figures S10A and S10C) and P60 (Figures S10B and S10D). These results indicate that CK1α deletion had no influence on apoptosis.

Next, the proliferation of POMC^+^ neurons was examined by POMC and EdU dual staining in the E12.5 hypothalamus before the massive reduction in their number. The proportions of EdU^+^POMC^+^ cells were not different between control and PKO mice (Figures 4G and 4H). To further determine how CK1α affected POMC neurons, ROSA^mT/mG^ reporter mice were crossed with control and PKO mice for cell lineage tracing, to label cre-mediated neuronal protrusions and recombination cells permanently and genetically. The neuronal-fiber density and GFP neuron numbers were not different between control and PKO mice (Figures S11A–S11C), which was consistent with the lack of apoptotic differences found at E15.5 and P60 (Figures 4A–4D). In short, neither neuronal architecture nor cellular survival was affected by CK1α deletion in Pomc-expressing cells.

The proportions of E15.5 (POMC^+^GFP^+^: 17.7% and POMC^−^GFP^+^: 82.3%) and P60 (POMC^+^GFP^+^: 25.4% and POMC^−^GFP^+^: 74.6%) ARC POMC^+^GFP^+^ neuron counts of Csnk1a1^fl/fl^;POMC^cre^; ROSA^mT/mG^ mice were significantly reduced relative to E15.5 (POMC^+^GFP^+^: 71.2% and POMC^−^GFP^+^: 28.8%) and P60 (POMC^+^GFP^+^: 81.4% and POMC^−^GFP^+^: 18.6%) POMC^cre^;ROSA^mT/mG^ mice (Figures 4I, 4K, 4L, and 4N), confirming our previous results (Figures 3A and 3B). However, no change was observed in the number of ARC GFP signals (Figures 4J and 4M). The density of neuronal fibers was significantly reduced in the PVN of adult Csnk1a1^fl/fl^;POMC^cre^;ROSA^mT/mG^ mice (Figures 4I–4Q, Table S3). In addition, POMC progenitor cells could differentiate NPY neurons, and so NPY *in situ* hybridization (ISH) was performed. No significant differences were found in NPY neuron counts in PKO hypothalamus ARCs at P7 and P60 (Figure S12A). Overall, our results demonstrate that CK1α is required to maintain normal levels of POMC expression in ARC neurons, thereby regulating energy homeostasis.

### Inhibition of notch signaling restores the phenotype of PKO mice

**Figure 5:**
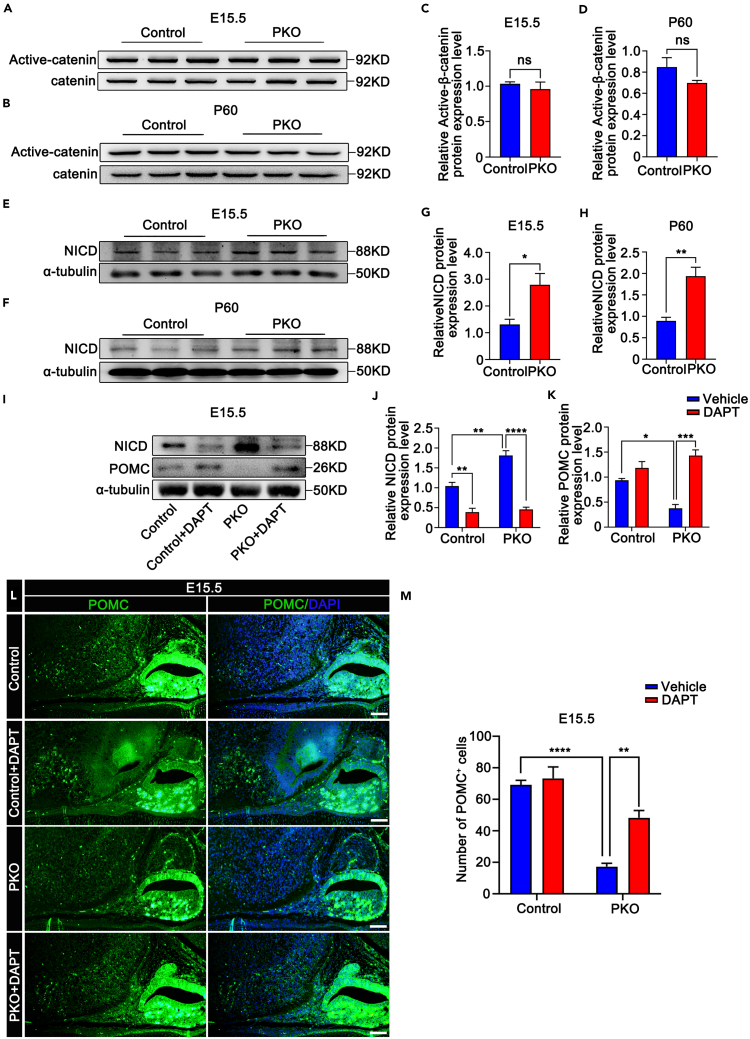
Notch signaling pathway inhibition restores the phenotype of PKO mice (A and B) Active β-catenin protein expression in control and PKO hypothalami at E15.5 (A) and P60 (B). (C and D) Quantification of active β-catenin protein in control and PKO hypothalami at E15.5 (C) and P60 (D) (n = 3, ns p > 0.05). Data are means ± SEM. (E and F) NICD and α-tubulin protein expression in control and PKO hypothalami at E15.5 (E) and P60 (F). (G and H) Quantification of NICD protein expression in control and PKO hypothalami at E15.5 (G) and P60 (H) (n = 3, ∗p < 0.05, ∗∗p < 0.01). Data are means ± SEM. (I) NICD and POMC protein expression in hypothalami of control and PKO mice injected with NaCl or 10 mg/kg DAPT at E15.5. (J and K) Quantification of NICD (J) and POMC (K) protein in control and PKO hypothalami at E15.5 (n = 3, ∗p < 0.05, ∗∗p < 0.01, ∗∗∗p < 0.001, ∗∗∗∗p < 0.0001). Data are means ± SEM. (L) POMC immunofluorescence staining in E15.5 control and PKO hypothalami injected with 10 mg/kg DAPT. Scale bars, 50 μm. (M) Numbers of POMC^+^ neurons in E15.5 control and PKO hypothalami after the injection of NaCl or 10 mg/kg DAPT (n = 4, ∗∗p < 0.01, ∗∗∗∗p < 0.0001). Data are means ± SEM. Control = Csnk1a1^fl/+^;POMC^cre^. PKO = Csnk1a1^fl/fl^;POMC^cre^.

As CK1α regulates biological processes through the Wnt/β-catenin pathway, we hypothesized that it regulates POMC expression through Wnt/β-catenin signaling. To test this hypothesis, we detected active β-catenin signaling using western blotting. No significant differences were observed in E15.5 (Figures 5A and 5C) and P60 (Figures 5B and 5D) PKO hypothalami compared with controls. Our data indicate that the Wnt/β-catenin pathway is unlikely to be the primary pathway regulating POMC expression.

Notch signaling negatively regulates POMC expression. Thus, we determined whether Notch signaling was activated in the hypothalamus of PKO mice using western blotting. NICD expression in E15.5 (Figures 5E and 5G) and P60 (Figures 5F and 5H) PKO hypothalami was increased 2.15- and 2.18-fold as compared to that in control hypothalami. If the regulation of Notch signaling by CK1α greatly influenced POMC expression, we predicted that such regulation may rescue PKO mouse phenotypes. To confirm this, the Notch signaling pathway inhibitor N-[N-(3,5-difluorophenacetyl)-*l*-alanyl]-S-phenylglycine *t*-butyl ester (DAPT) was injected into pregnant dams intraperitoneally from E12.5 until E15.5 and injected intracerebroventricular (i.c.v.) into the third ventricle on P60 male control and PKO mice. Hypothalami were harvested at E15.5 and P60. Western blotting showed that DAPT decreased NICD protein expression 1.73- to 0.44-fold in E15.5 PKO hypothalami (Figures 5I and 5J) and decreased NICD protein expression 1.40- to 0.36-fold in P60 PKO hypothalami (Figures S13A and S13B). POMC protein levels recovered significantly from 39.8 to 152.6% in E15.5 PKO hypothalami (Figures 5I and 5K) and from 30.7 to 189.0% in P60 PKO hypothalami (Figures S13A and S13C) compared with that of controls. Consistently, NICD protein suppression resulted in an increase in the number POMC^+^ neurons from 17 ± 2 to 48 ± 5 in E15.5 PKO hypothalami (Figures 5L and 5M) and from 11 ± 2 to 29 ± 4 in P60 PKO hypothalami (Figures S13D and S13E). In summary, CK1α regulates the development of murine POMC neurons via NICD-POMC signaling.

## Discussion

Our previous work suggested that CK1α was involved in the development of murine testis^28^ and ovaries^32^; however, its role in neural development and control of energy homeostasis remains elusive. We found that CK1α deletion in POMC neurons led to food intake, metabolic defects, and increased body weight, which were associated with a decrease in POMC-positive cell counts. Our data show that CK1α is required to maintain normal levels of POMC expression in ARC neurons mediated by NICD-POMC signaling during embryonic life.

Neuron numbers are regulated by their migration, proliferation, death, and differentiation.^33^ In the murine hypothalamic ARC, *Pomc* mRNA was first detected at E10.5. Recent studies confirm that POMC progenitor cells differentiate to terminal phenotypes at E15.5 using genetic lineage tracing. As early as E15.5, we found a large reduction in the number of POMC-positive cells in PKO mice, suggesting the impairment of neurogenesis. Compared with the controls, the distribution of POMC neurons in adult PKO mice ARCs was similar, suggesting that POMC cell migration was not affected. Alternatively, the large reduction in the number of POMC neurons may be caused by increased cell death, which would be consistent with the results of previous reports, demonstrating that CK1α knockout leads to cell apoptosis in the intestinal epithelium.^34^ Cell lineage tracing proved that these neurons survived in PKO mice. Consistently, we found no significant difference in ARC apoptotic signals between PKO and control mice. Most Cre recombinant neurons in PKO mice lacked POMC immunoreactivity, suggesting that CK1α ablation in POMC progenitor populations disrupted their ability to express POMC. Our results show that CK1α is essential for POMC expression in ARC neurons.

We found that CK1α deletion did not affect POMC expression via Wnt/β-catenin signaling. As a Wnt/β-catenin signaling negative regulator,^35^ CK1α deletion causes the activation of β-catenin^18^ in the intestinal epithelium,^34^ keratinocytes,^36^ and spermatogonia.^28^ Recent reports showed that knockdown of β-catenin by CaMKIIα-iCre decreased the numbers of POMC neurons,^37^ which is not consistent with our results. However, we found that Wnt/β-catenin signaling was not affected in POMC neurons. Thus, the Wnt/β-catenin pathway may not regulate POMC expression.

Finally, we showed that CK1α regulates POMC expression via NICD-POMC signaling. Corroborating the results of earlier reports on the inhibitory effect of Notch on hypothalamic neuronal development,^38^ we detected a significant increase in NICD protein expression in CK1α knockout mice, along with a significant decrease in POMC neuron counts. In addition, *in-vivo* DAPT treatment restored POMC expression. These results provide strong evidence that CK1α regulates POMC expression through the Notch signaling pathway.

Collectively, our findings confirm a mechanism in the regulation of POMC expression that involves CK1α and NICD/Pomc signaling. CK1α conditional deletion in mouse POMC neurons caused a decrease in their numbers and an increase in NICD protein levels, which subsequently resulted in a decrease in α-MSH peptide expression, eventually leading to obesity in PKO mice. Our data confirm a regulatory mechanism of POMC expression during embryogenesis that is critical to our understanding of the pathogenesis of obesity.

### Limitations of the study

Our present study suggests that CK1α plays crucial roles in the development of POMC neurons. There are a few limitations to our study. First, CK1α conditional knockout results in the decrease of POMC neurons during embryonic development, however, the function of CK1α in mature POMC neurons after birth requires further investigation using TAM-inducible CK1α conditional knockout mice. Furthermore, we have shown that CK1α regulates POMC expression via NICD-POMC signaling, however, the link between CK1α and NICD is unknown. This awaits further molecular genetic investigation.

## STAR★Methods

### Key resources table

**Table:** 

REAGENT or RESOURCE	SOURCE	IDENTIFIER
**Antibodies**		

Anti-rabbit CK1α (1/1000 WB, 1/200 IF, For antibody specificity validation test)	Abcam	Cat# ab64939 RRID: AB_2934225
Anti-chicken CK1α (1/250 IF, be tested for antibody specificity in Figure S14)	Novus Biologicals	Cat# NBP2-50030 RRID: AB_2934242
Anti-rabbit CK1α (1/200 IF, For antibody specificity validation test)	Proteintech	Cat# 55192-1-AP RRID: AB_11183034
Anti-rabbit α-tubulin (1/10000 WB)	Beyotime	Cat# AF0001 RRID: AB_2922414
Anti-goat POMC (1/200 IF,1/1000 WB, be tested for antibody specificity in Figure S14)	Abcam	Cat# ab32893 RRID: AB_777375
Anti-mouse ACTH (1/100 IF, For antibody specificity validation test)	Santa Cruz Biotechnology	Cat# sc-57021 RRID: AB_785253
Anti-rabbit C-Caspase3 (1/200 IF)	Cell Signaling Technology	Cat# 9661 RRID: AB_2341188
Anti-rabbit Active β-catenin (1/1000 WB)	Cell Signaling Technology	Cat# 8814 RRID: AB_11127203
Anti-rabbit anti-β-catenin (1/1000 WB)	Cell Signaling Technology	Cat# 8480 RRID: AB_11127855
Anti-mouse Stat3 (1/1000 WB)	Cell Signaling Technology	Cat# 9139 RRID: AB_331757
Anti-rabbit Phospho-Stat3 (1/1000 WB)	Cell Signaling Technology	Cat# 9145 RRID: AB_2491009
Anti-rabbit NICD (1/500 WB)	Wanlei Bio	Cat# WL03097a RRID: AB_2934244
Anti-rabbit α-MSH (1/1000 IF)	Abcam	Cat# ab53568 RRID: AB_881127
Anti-mouse TSHb (1/200 IF)	Hangzhou HuaAn Biotechnology	Cat# EM1706-3 RRID: AB_2934247
Anti-rabbit GH (1/100 IF)	Abcam	Cat# ab155276 RRID: AB_2934251
Anti-rabbit LepR (1/1000 WB)	Hangzhou HuaAn Biotechnology	Cat# ET1704-44 RRID: AB_2934245
Cy3 Donkey Anti-Chicken (1/200 IF)	Jackson ImmunoResearch Labs	Cat# 703-165-155 RRID: AB_2340363
Cy2 Donkey Anti-Rabbit (1/200 IF)	Jackson ImmunoResearch Labs	Cat# 711-225-152 RRID: AB_2340612
488 Bovine Anti-Goat (1/200 IF)	Jackson ImmunoResearch Labs	Cat# 805-545-180 RRID: AB_2340883
Cy5 Donkey Anti-Goat (1/200 IF)	Jackson ImmunoResearch Labs	Cat# 705-175-147 RRID: AB_2340415
Cy3 Donkey Anti-Goat (1/200 IF)	Jackson ImmunoResearch Labs	Cat# 705-165-003 RRID: AB_2340411
488 Donkey Anti-Mouse (1/200 IF)	Jackson ImmunoResearch Labs	Cat# 715-545-150 RRID: AB_2340846
Goat Anti-Rabbit (1/10000 WB)	Jackson ImmunoResearch Labs	Cat# 111-005-003 RRID: AB_2337913
Goat Anti-Mouse (1/10000 WB)	Jackson ImmunoResearch Labs	Cat# 115-005-003 RRID: AB_2338447
Donkey Anti-Goat (1/10000 WB)	Jackson ImmunoResearch Labs	Cat# 705-005-003 RRID: AB_2340384

**Chemicals, peptides, and recombinant proteins**		

5-ethynyl-2′-deoxyuridine (EdU)	Beyotime	Cat# C0081S
α-MSH	Gene Script Inc.	Cat# RP10644
Leptin	Gene Script Inc.	Cat# Z03158
RIPA	Beyotime	Cat# P0013B
PMSF	Sangon Biotech	Cat# A610425
DAPI	Beyotime	Cat# C1002
DAPT	MCE	Cat# HY-13027

**Experimental models: Organisms/strains**		

Csnk1a1 flox/flox mice	The Jackson Laboratory	Cat# 025398
Pomc1-Cre	The Jackson Laboratory	Cat# 005965
ROSAmT/mG reporter mice	The Jackson Laboratory	Cat# 007576
C57BL/6 mice	Yangzhou university	N/A

### Resource availability

#### Lead contact

Further information and requests for resources and reagents should be directed to and will be fulfilled by Sheng Cui (cuisheng@yzu.edu.cn).

#### Materials availability

This study did not generate new unique reagents and all materials in this study are commercially available.

### Experimental model and subject details

#### Animals

All mice were housed on a 12-h/12-h light/dark cycle with CD in a 22–24°C environment, unless indicated otherwise. All mice were maintained in the C57BL/6 and 129/SvEv mixed background, and all animal experiments followed protocols approved by the Institutional Animal Care and Use Committee of Yangzhou University. *Csnk1a1*^*Flox*^ mice were genotyped with the following PCR primers: forward, 5′-GGTCCTCCAGATTCCACAGT-3′, reverse, 5′-TAGGGACCAAAGACGACCTG-3′. *POMC*^*cre*^ mice are genotyped with cre PCR primers: forward, 5′-GCGGTCTGGCAGTAAAAACTATC-3′, reverse, 5′-GTGAAACAGCATTGCTGTCACTT-3′ and with control PCR primers: forward, 5′-CTAGGCCACAGAATTGAAAGATCT-3′, reverse, 5′-GTAGGTGGAAATTCTAGCATCATCC-3′. ROSA^mT/mG^ mice were genotyped with wild PCR primers: forward, 5′- CTCTGCTGCCTCCTGGCTTCT-3′, reverse, 5′-CGAGGCGGATCACAAGCAATA-3′ and with mutant PCR primers: forward, 5′-CTCTGCTGCCTCCTGGCTTCT-3′, and reverse, 5′- TCAATGGGCGGGGGTCGTT-3′.

The plug day was considered as embryonic day E0.5. On E12.5 and E15.5, female pregnant mice were sacrificed to collect embryos for immunohistochemistry, real-time PCR, and western blotting. The birth day was considered as postnatal day 1. E15.5 to P60 mice were performed for CK1α expression in hypothalamic development. 5-week-old and 12-week-old male mice were performed for glucose tolerance test and insulin tolerance test. 12-week-old male mice were performed for indirect calorimetry, body composition analysis, leptin function and sensitivity, α-MSH treatment and histology. Unless above noted, male P60 mice were used for other experiments.

### Method details

#### Physiological measures

Mice were housed individually to measure food consumption. Body weight and food intake were calculated at 8:00 a.m. daily for 5 days to calculate mean 5-day intakes. For the glucose tolerance test, mice were injected with 1.5 g/kg glucose after overnight fasting. At 0, 15, 30, 60, 90, and 120 min post-injection, tail blood glucose concentration (mg/dl) was measured using a handheld glucometer (One Touch). For the insulin tolerance test, mice were injected with 0.5 U/kg insulin after having fasted for 4 h beginning at 9:00 a.m. At 0, 30, 60, 90, and 120 min post-injection, blood glucose levels were measured from tail blood as described above.

#### Real-time PCR

Mouse hypothalamus and pituitary RNAs were extracted using TRIzol reagent (TaKaRa). HiScript Q RT SuperMix for qPCR (+gDNA wiper; Vazyme; R123-01) was performed to synthesize complementary DNA. ChamQ SYBR qPCR Master Mix (Vazyme; Q311-02/03) was performed to measure gene expression levels in the ABI StepOnePlus™ Sequence Detection System (Applied Biosystems). The delta–delta Ct method was used for quantifying gene expressions with *Gapdh* for normalization. The primers are listed in Table S4.

#### Western blotting

Hypothalamic protein extracts were manually homogenized in radioimmunoprecipitation assay buffer (RIPA, Beyotime Biotechnology) containing 1 mM phenylmethyl sulfonyl fluoride (PMSF, Sangon Biotech). According to the manufacturer’s instructions, hypothalamic protein concentrations were examined using the bicinchoninic acid assay reagent (JiangSu CoWin Biotech). Protein quantification was performed using the Quantity One software (Bio-Rad).

#### Immunohistochemistry

For E12.5 and E15.5 hypothalami sagittal sections (Figures 1E, 3A, 4A, and 4G) and pituitaries sections, hypothalami and 4Gpituitaries were fixed overnight in 4% PFA, then dehydrated, embedded in paraffin wax, and sectioned (5 μm). The entire E12.5 and E15.5 ARCs were cut into 25 and 50 pieces, respectively. Every five sections were stained, and cell counts were conducted on five and ten sections per brain, respectively. Immunohistochemistry of hypothalamus sagittal sections was performed with the primary antibodies: anti-CK1α (1:250, Novus Biologicals) and anti-POMC (1:200, Abcam) overnight at 4°C. Immunohistochemistry of pituitary sections was performed with the primary antibodies: anti-TSHb (1:200, Huabio), anti-GH (1:100, Abcam), and anti-POMC (1:200, Abcam) overnight at 4°C. The cell counts in the figure represent the average number of cells for each section.

For E15.5 and adult coronal sections (Figures 1F, 2A, 4B, 4I, 4L, 4O, S1, S10, and S12D), mice were anesthetized using pentobarbital sodium and perfused with 50 mL 0.9% NaCl and 50 mL 4% PFA transcardially. Hypothalami were fixed overnight in 4% PFA, settled in 30% sucrose, embedded in O.C.T., and frozen. Then, 20-μm-thick frozen sections were cut and placed immediately on slides using a freezing microtome. The entire E15.5 and adult ARC were cut into 15 and 80 pieces, respectively. Every five sections were stained, and cell counts were conducted on three and 16 sections per brain. Then sections were thawed for 15 min at 37°C, washed in PBS, perforated in 0.5% Triton-X100 for 10 min, and blocked in 1% BSA for 60 min. Finally, primary antibodies [anti-CK1α (1:250, Novus Biologicals) and anti-POMC (1:200, Abcam)], secondary antibodies, and DAPI were used for staining. The cell counts in the figure represent the average number of cells for each section.

For α-MSH chromogenic immunostaining in the PVN (Figure 3H), mice were anesthetized using pentobarbital sodium and perfused with 50 mL 0.9% NaCl and 50 mL 4% PFA transcardially. Hypothalami were fixed overnight in 4% PFA, settled in 30% sucrose, embedded in O.C.T., and frozen. Then, 20-μm-thick frozen sections were thawed for 15 min at 37°C, washed in PBS, perforated in 0.5% Triton-X100 for 10 min, and blocked in 1% BSA for 60 min. Finally, primary antibody [anti-α-MSH (1:1000, Abcam)] and horseradish peroxidase goat anti-rabbit secondary antibody were used for staining. All sections were visualized by using diaminobenzidine-H_2_O_2_. The α-MSH integrated density in the PVN was calculated using the ImageJ software.

#### Indirect calorimetry and body composition analysis

To measure indirect calorimetry, control (Csnk1a1^fl/+^;POMC^cre^) and PKO mice were acclimatized in experimental cages for 24 h and then monitored for 72 h and analyzed according to the 72 h experimental data using a TSE Phenomaster.

To measure body composition (lean and fat mass), mice were assayed using EchoMRI.

#### EdU labeling

For EdU labeling, 50 mg/kg of 5-ethynyl-2′-deoxyuridine (EdU, Beyotime Biotechnology, C0081S) in ∼200 μL of sterile saline was injected daily from E10.5–E12.5 intraperitoneally into pregnant dams. Dams were euthanized and brains of E12.5 embryos were processed for immunohistochemistry.

#### Plasma leptin and hypothalamic α-MSH level assessment

Plasma leptin and hypothalamic α-MSH protein contents were analyzed using ELISA (Ruixin Biotechnology) according to the manufacturer’s instructions. Each mouse serum sample was prepared for leptin ELISA and each mouse hypothalamic tissue homogenate was prepared for α-MSH ELISA. Firstly, standard and sample holes were set up, and different concentrations of 50 μL standard products were added into the standard holes. Blank and sample holes were arranged. We added 40 μL of sample dilution and another 10 μL of sample to the enzyme label-coated plate to test the sample. Then, the sample was added to the bottom of the hole of the enzyme label plate. After sealing the plate with sealing plate film, it was incubated at 37°C for 30 min. Then, 1x washing solution was prepared and each hole was washed with 1x washing solution, which was repeated five times. Next, 50 μL enzyme-labeled reagent was added to each well and the incubation and washing operations were repeated. Then, 50 μL of color developing agents A and B was added to each well to display the color for 15 min at 37°C. Finally, 50 μL of termination solution was added to each well to terminate the reaction. The absorbance (OD value) of each hole was measured in sequence with blank air conditioner zero and a wavelength of 450 nm.

#### Leptin function and sensitivity

Mice were individually housed for 1 week, then intraperitoneally injected with leptin (1.5 mg/kg) at 7:00 a.m. and 7:00 p.m. for 3 days. Food intake and body weight gain were measured each day at 7:00 a.m. For leptin sensitivity experiments, PKO and control (Control = Csnk1a1^fl/+^;POMC^cre^) mice were injected intraperitoneally with vehicle or leptin (1.5 mg/kg). Hypothalami were collected for p-STAT3 (Tyr705) western blotting 45 min after injection.

#### α-MSH treatment

Before the beginning of the dark cycle, 1 mg/kg α-MSH peptide^39^ (Gene Script Inc., RP10644) was injected intraperitoneally for 3 days. Body weight gain and food intake were then measured each day at 7:00 a.m.

#### Histology

Mouse BAT, iWAT, eWAT, and liver were fixed in 4% paraformaldehyde (4% PFA), dehydrated, embedded in paraffin, sectioned (5 μm), and immersed with hematoxylin and eosin (H&E). For Oil Red O staining, 10-μm-thick frozen liver sections were incubated with 0.5% Oil Red O solution.

#### *In-situ* hybridization (ISH)

*In situ* hybridization probe was performed. The primers designed for NPY were hybridized with the following PCR primers: forward,5′- TAATACGACTCACTATAGGGCTAGGTAACAAGCGAATGG-3′, reverse,5′- AATTAACCCTCACTAAAGGGTTGGTGGGACAGGCAGAC-3′. The primers designed for POMC were hybridized with the following PCR primers: forward,5′- TAATACGACTCACTATAGGGTGCCGAGATTCTGCTAC-3′, reverse,5′-AATTAACCCTCACTAAAGGGCCGTTTCTTGCCCACC-3′. NPY and POMC gene sequences were amplified by PCR, the products were purified, recovered, and the DNA concentration was measured. NTP-s (Mix), RNasin, 5 x buffer, and T3 enzyme were used for transcription. DNase Ⅰ was used to digest DNA at 37°C for 15 min, and the reaction was stopped by adding 1 μL of 0.2 M EDTA. Then, 4M LiCl and ethanol were used for RNA precipitation.

Mouse hypothalami were collected and placed in 4% DEPC-PFA and fixed overnight at 4°C and placed in DEPC-30% sucrose solution overnight at 4°C until the tissue settled to the bottom of the tube. Mouse hypothalami was immersed with O.C.T embedding agent and fixed in liquid nitrogen and stored at −80°C or sliced directly. The embedded mouse hypothalami were fixed on the cutter head of the frozen microtome, and the slice thickness was adjusted to 20 μm. After slicing, we dried the slices into the oven and added 4% PFA to fix for 10 min. Then, 5 μg/mL Proteinase K solution was added to sections for 10 min. To reduce the non-specific coloring of hybridization, acetylation solution was used for 10 min. After the slides had been cleaned by DEPC-PBS, the prehybridization solution was added and incubated at room temperature for 4 h. The hybrid solution was added with 2 ng/μL NPY probe and placed in the hybrid wet box and incubated at 65°C for 16 h. Slices of mouse hypothalami were immersed in 5×SSC and 0.2×SSC solution at 65°C incubated for 30 min and sealed with 5% bovine serum for 1 h. Digoxin antibody (1:1000) labeled with alkaline phosphatase was added and incubated overnight at 4°C. NBT/BCIP staining solution was added to mouse hypothalami slices, and the termination of reaction was determined according to the observed color development.

#### Image analysis

Images were obtained using a Leica SP8 confocal system with a 20X oil immersion lens and analyzed using the ImageJ software. The average number of ARC GFP cells and density of fibers were analyzed in three hemisections. For the quantitative analysis of cell number, the numbers of POMC^+^ cells, Pomc^+^GFP^+^ cells and NPY^+^ cells were manually counted using the ImageJ software for the quantitative analysis of cell number. Only cells with a DAPI nucleus were counted in our analysis.

For the quantitative analysis of fiber density, the relative density of α-MSH^+^ fibers and POMC^+^ fibers in PVN were analyzed using the ImageJ software. To separate the labeled fibers from the background, ImageJ was used to compensate for the difference in fluorescence intensity, and each image plane was binarized. Then, the combined intensity of each image was calculated and summed.

#### Notch inhibition studies

DAPT, an inhibitor of the Notch signaling pathway, was purchased from MCE (MedChemExpress); 10 mg/kg DAPT^40^ was administered intraperitoneally every 24 h from E12.5 to E15.5.

For DAPT Intracerebroventricular (i.c.v.) injection and treatments, DAPT was dissolved in 0.9 NaCl with 5% DMSO to prepare 8.3 mg/mL concentration. I.c.v. injection was carried out on P60 male control (Csnk1a1^fl/+^;POMC^cre^) and PKO mice. DAPT solution was stereotactically injected into the third ventricle immediately. The stereotactic injections into the third ventricle were performed at midline 0 mm, ± 0.8 mm posterior from bregma, and −4.5 mm depth from the bregma. Control (Csnk1a1^fl/+^;POMC^cre^) and PKO mice were injected with 2 μl of either vehicle (aCSF) or 0.03 mg/kg DAPT. Then, 6 h after injection, the hypothalamus was dissected for western blotting or perfused for immunohistochemistry studies.

### Quantification and statistical analysis

Data are expressed as means ± SEM. All analyses of real-time PCR and imaging data were performed with at least three independent replicates. Prism 7 (GraphPad Software) was used for all analyses. When two independent groups were compared, a two-tailed Student’s *t*-test was performed. When multiple independent groups were compared, two-way analysis of variance (ANOVA) followed by Tukey’s multiple comparisons test was performed. Statistical significance was set at P < 0.05.

## Data Availability

• This paper does not report original code. • The data reported in this paper will be shared by the lead contact upon request. • The additional information required to reanalyze the data reported in this paper is available from the lead contact upon request.
