# Supplementary material for: Casein kinase 1α is required to maintain murine hypothalamic pro-opiomelanocortin expression
Source: iScience. 2023 Apr 14;26(5):106670. doi: 10.1016/j.isci.2023.106670 (PMC10165255; doi:10.1016/j.isci.2023.106670)
Supplement: Document S1. Figures S1–S14 and Tables S1–S4 [file mmc1.pdf]

iScience, Volume *26*

Supplemental information

Casein kinase 1α is required to maintain murine hypothalamic pro-opiomelanocortin expression

Chenyang Lu, Jinglin Zhang, Bingjie Wang, Qiao Gao, Kezhe Ma, Shaona Pei, Juxue Li, and Sheng Cui

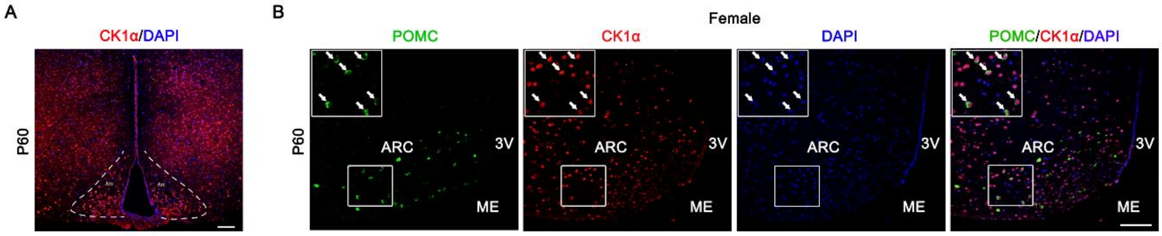

Figure S1. CK1α is expressed in P60 female hypothalami. Related to Figure 1.
(A) Immunofluorescence detection of CK1α. Scale bar, 100 μm. (B) Immunofluorescence detection of CK1α (red) and Pomc (green). Arrows indicate representative CK1α$^+$ Pomc$^+$ cells. DNA was stained with DAPI. Scale bar, 50 μm.

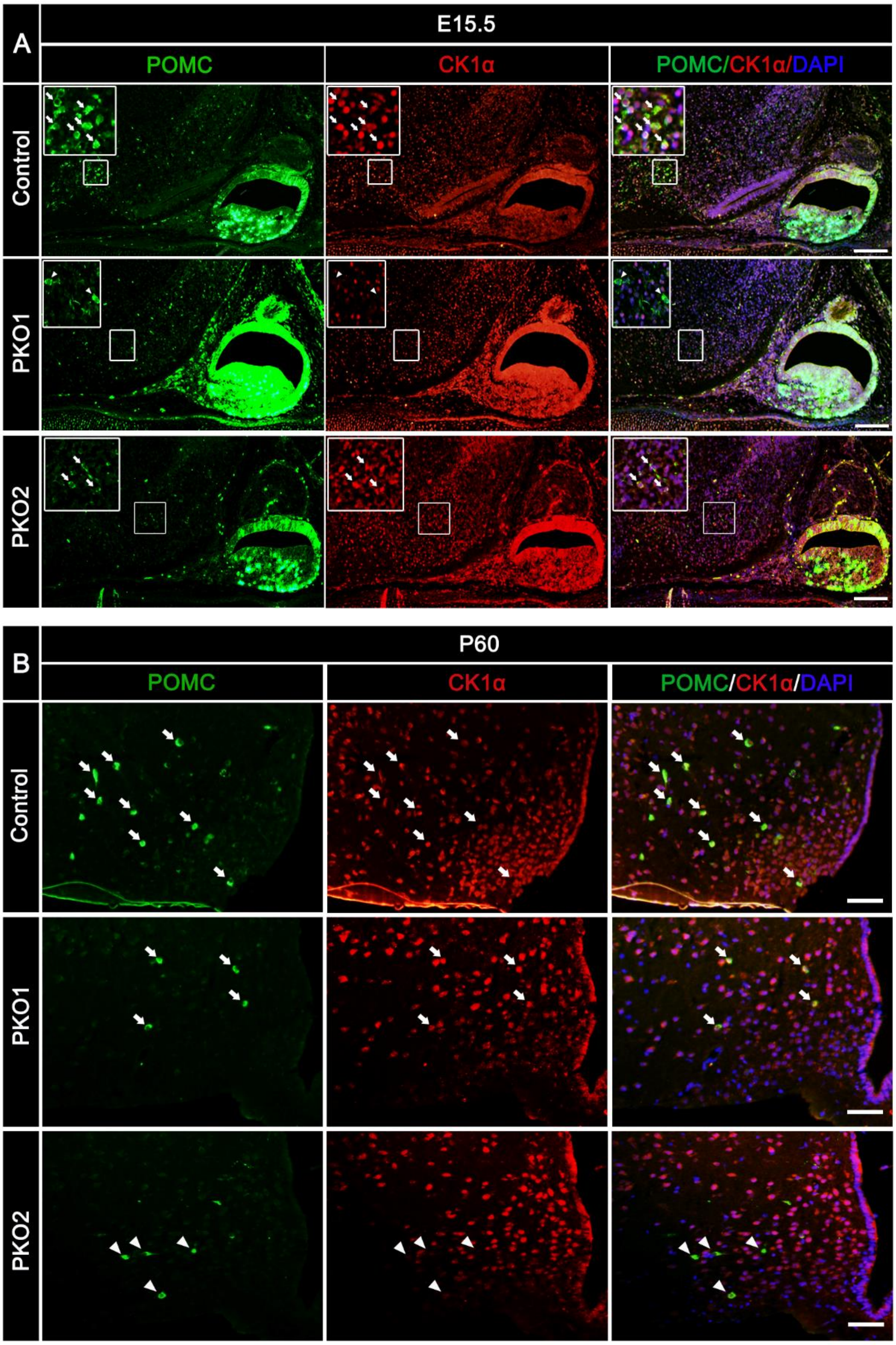

Figure S2. CK1α was still expressed in some remaining POMC neurons in PKO mice. Related to Figure 2.

(A,B) Double immunofluorescence staining for POMC and CK1α in control and PKO hypothalami at E15.5 (A) and P60 (B). Scale bars, 50 μm.

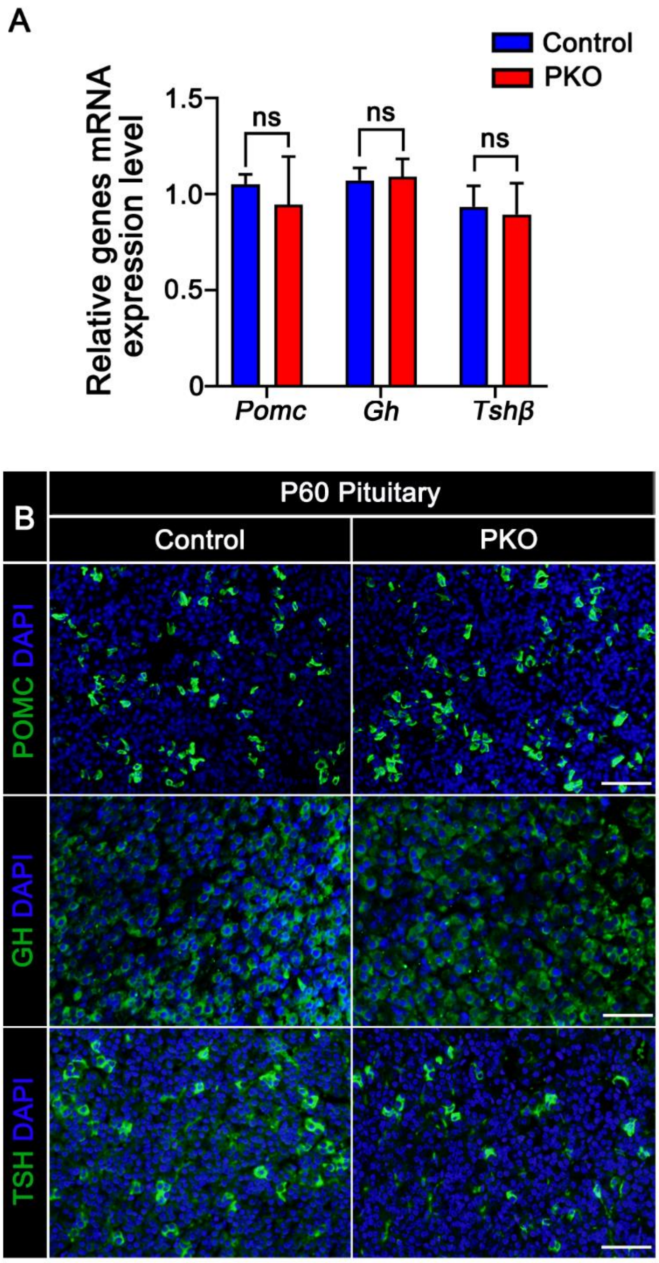

Figure S3. Deletion of CK1α does not cause pituitary defects. Related to Figure 2.

(A) Real-time PCR quantification of *Pomc, Gh,* and *Tshβ* expression in control and PKO hypothalami at P60 (n = 5, ns P > 0.05). (B) Immunofluorescence detection of POMC, GH, and TSHβ in P60 mouse pituitary. DNA was stained with DAPI. Scale bars, 50 μm. Control = $Csnk1a1^{fl/+}$;$POMC^{cre}$. PKO = $Csnk1a1^{fl/fl}$;$POMC^{cre}$.

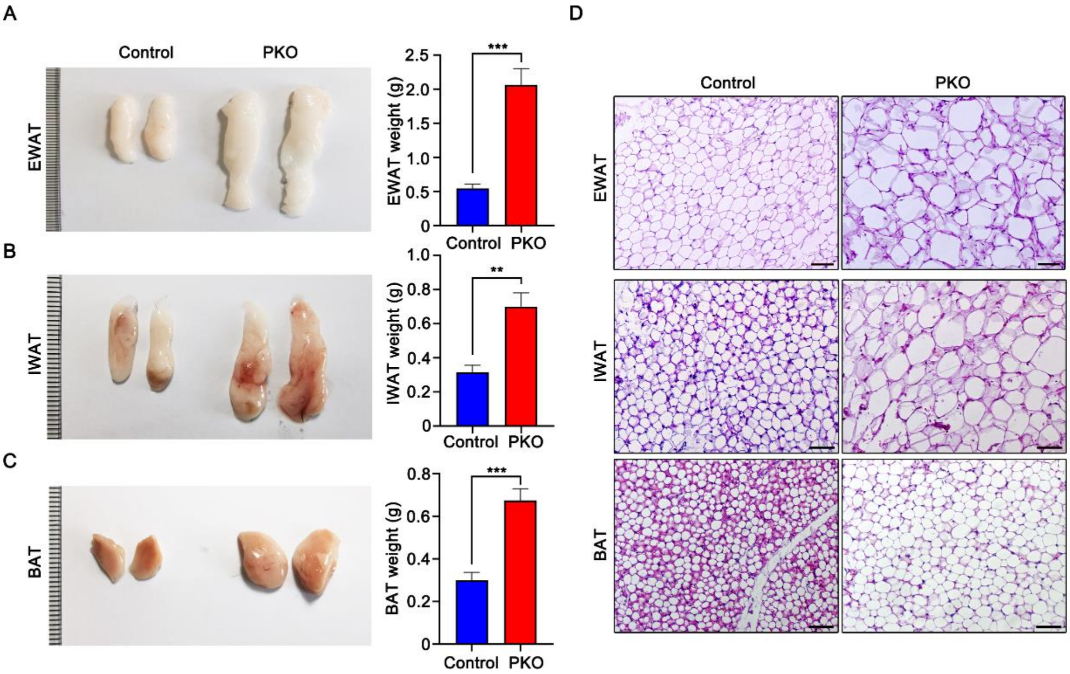

Figure S4. Deletion of CK1α in POMC neurons causes obesity. Related to Figure 2.
(A-C) Representative photographs and weight of eWAT (A), iWAT (B), and BAT (C) from 12-week-old control and PKO mice. (n = 5, ** $P < 0.01$, *** $P < 0.001$). D. Representative H&E staining of eWAT, iWAT, and BAT from control and PKO mice. Scale bars, 50 μm. Control = Csnk1a1$^{fl/+}$;POMC^{cre}. PKO = Csnk1a1$^{fl/fl}$;POMC^{cre}.

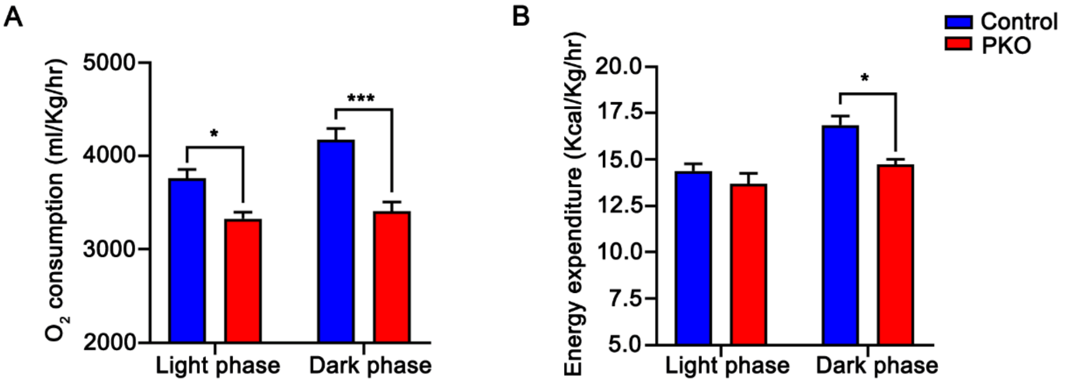

Figure S5. PKO mice are obese owing to reduced energy expenditure. Related to Figure 2.

(A) Daily oxygen consumption and (B) energy expenditure in control and PKO mice (n = 5, *P < 0.05, ***P < 0.001). Control = Csnk1a1$^{fl/+}$;POMC^{cre}. PKO = Csnk1a1$^{fl/fl}$;POMC^{cre}.

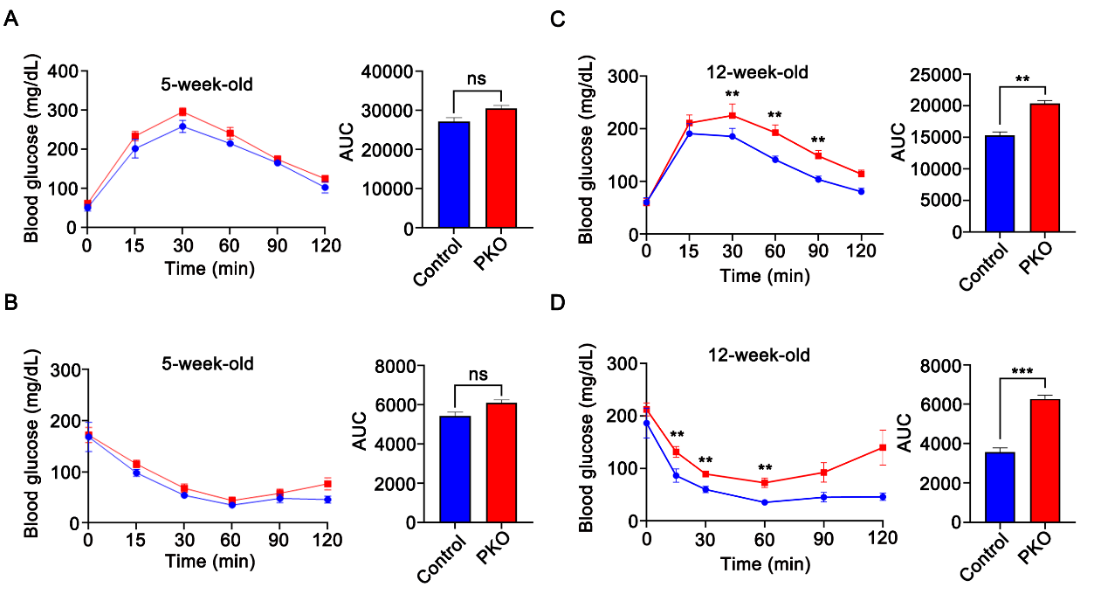

Figure S6. Deletion of CK1α in POMC neurons causes impaired glucose and insulin tolerance in 12-week-old mice. Related to Figure 2.

(A,C) Glucose tolerance in control and PKO mice at an age of 5 weeks (n = 5) and 12 weeks (n = 8). (B,D) Insulin tolerance in control and PKO mice at an age of 5 weeks (n = 5) and 12 weeks (n = 8) (ns $P > 0.05$; **$P < 0.01$; ***$P < 0.001$). Control = Csnk1a1$^{fl/+}$;POMC^{cre}. PKO = Csnk1a1$^{fl/fl}$;POMC^{cre}.

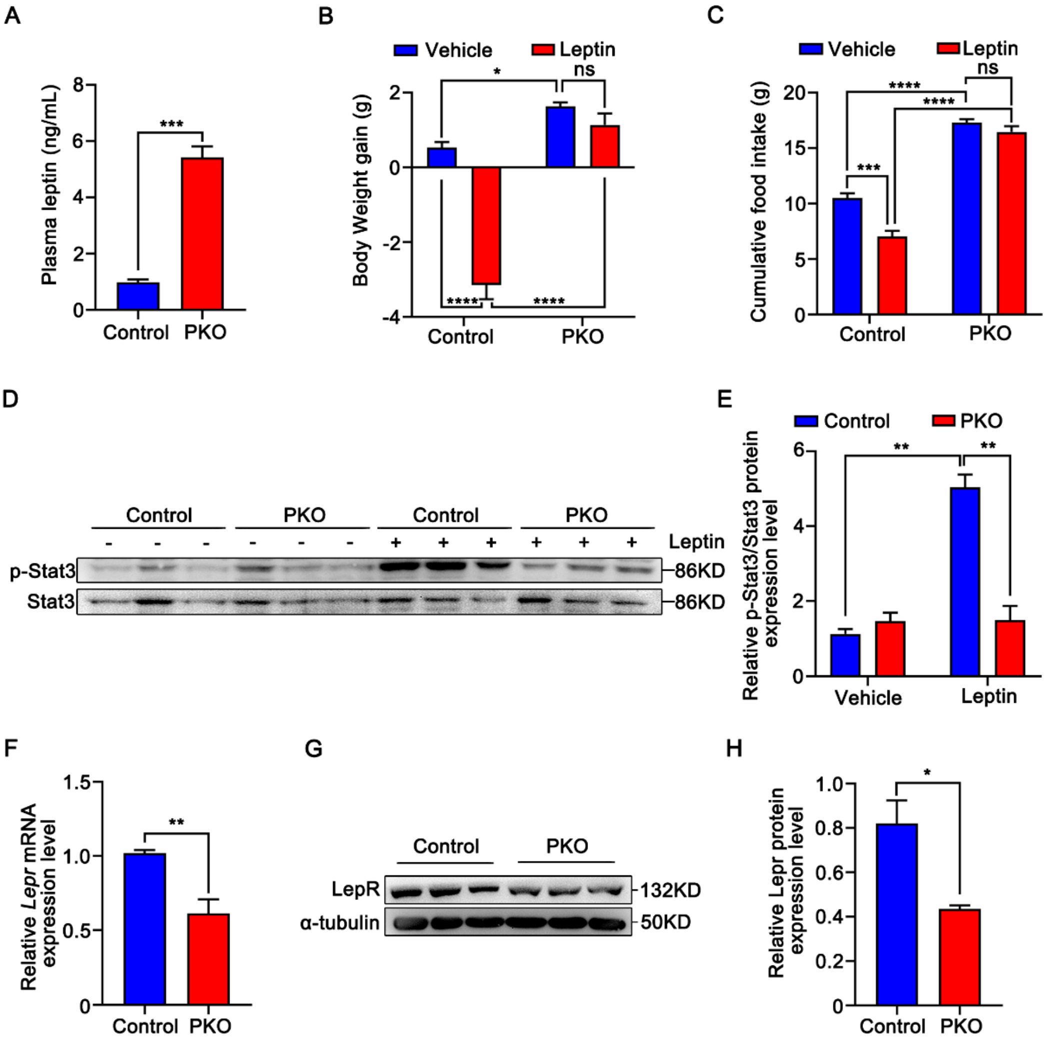

Figure S7. PKO mice develop leptin resistance. Related to Figure 2.

(A) Plasma leptin content in male control and PKO mice at 12 weeks of age (n = 5, *** P < 0.001). (B,C) Body weight gain (B) and cumulative food intake (C) in 12-week-old male control and PKO mice after 3 days of leptin injection (n = 4, ns P >0.05, * P < 0.05, ** P < 0.01, *** P < 0.001). (D,E) Expression of p-STAT3 (Tyr705) and STAT3 in hypothalami of 12-week-old control and PKO mice 45 min after intraperitoneal injection of vehicle or leptin (1.5 mg/kg; n = 3, ** P < 0.01). Data are means ± SEM. (F) Real-time PCR quantification of *Lepr* expression in control and PKO hypothalami at P60 (n = 4, **P < 0.01). (G) Western blotting analysis of Lepr protein expression in control and PKO hypothalami at P60 (n = 3, *P < 0.05). Data are means ± SEM. Control = Csnk1a1$^{fl/+}$;POMC^{cre}. PKO= Csnk1a1$^{fl/fl}$;POMC^{cre}.

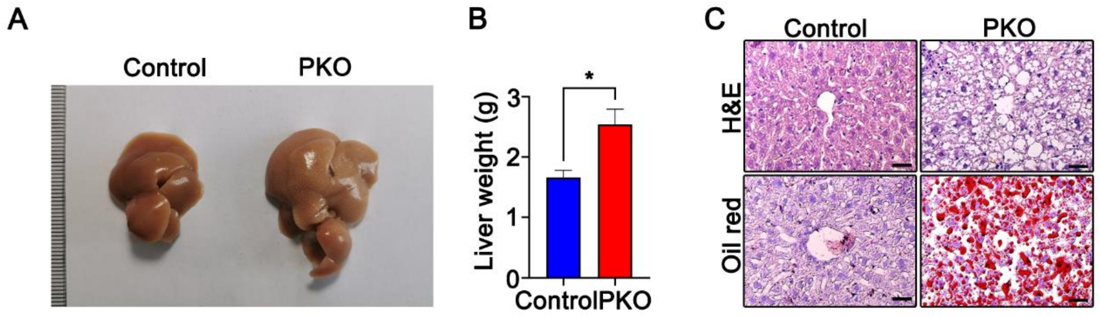

Figure S8. Deletion of CK1α causes hepatic steatosis. Related to Figure 2.

(A,B) Representative photographs (A) and weight (B) of the liver of 12-week-old control and PKO mice (n=5, *P <0.05). (C,D) Images of H&E- and Oil-red O-stained sections of 12-week-old control and PKO mice. Scale bar, 50 μm. Control = Csnk1a1$^{fl/+}$;POMC^{cre}. PKO = Csnk1a1$^{fl/fl}$;POMC^{cre}.

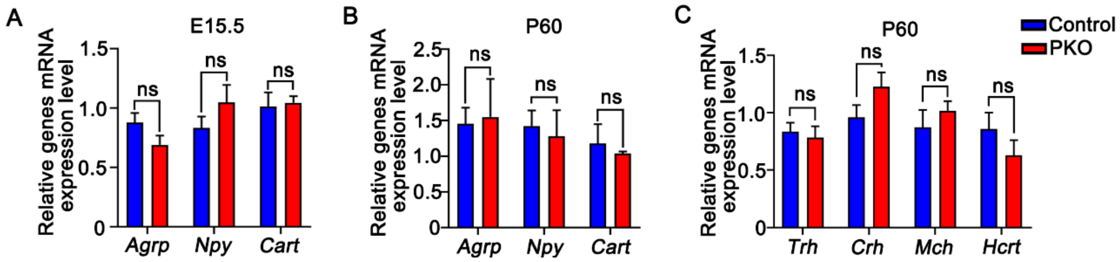

Figure S9. CK1α deletion does not affect the expression of *Agrp, Npy, Cart, Mch, Trh, Crh,* and *Hcrt* mRNAs. Related to Figure 3.

(A,B) Real-time PCR quantification of *Agrp*, *Npy*, and *Cart* mRNA expression in E15.5 (A) and P60 (B) mice (n = 6, ns P > 0.05). (C) Real-time PCR quantification of *Trh*, *Crh*, *Mch,* and *Hcrt* mRNA expression in P60 mice hypothalamus (n = 5, ns P > 0.05).

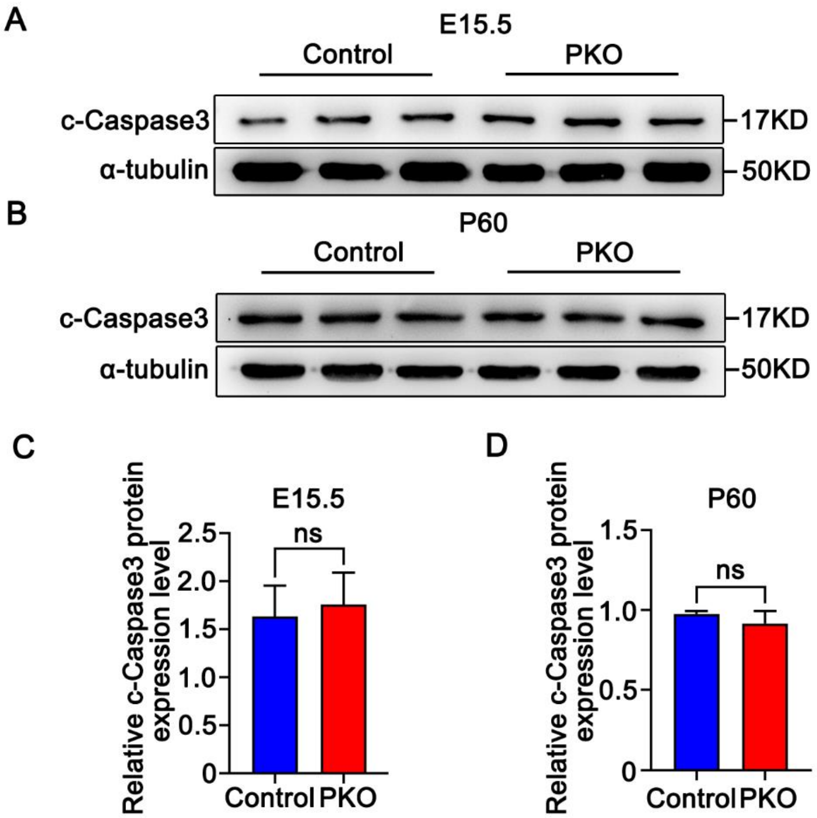

Figure S10. CK1α deletion has no influence on apoptosis. Related to Figure 4.

(A,B) Western blotting images of c-Caspase3 protein in the hypothalami of control and PKO mice at E15.5 (A) and P60 (B). (C,D) Quantification of c-Caspase3 protein level in the hypothalami of control and PKO mice at E15.5 (C) and P60 (D) (n = 3, ns P > 0.05). Control = Csnk1a1$^{fl/+}$;POMC^{cre}. PKO= Csnk1a1$^{fl/fl}$;POMC^{cre}.

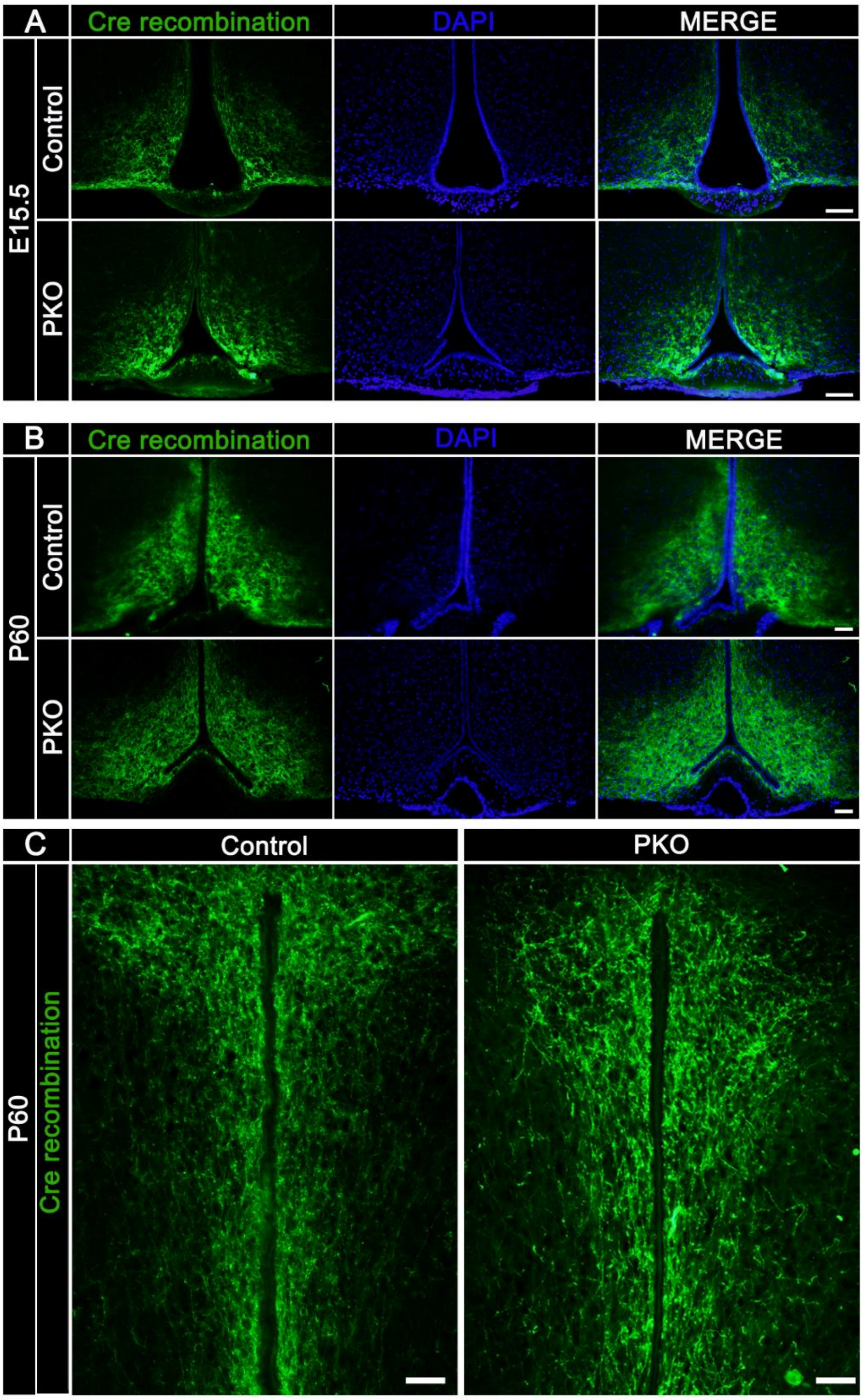

Figure S11. CK1α deletion has no influence on the neuronal-fiber density and GFP neuron numbers. Related to Figure 4.

(A,B) Representative micrographs of POMC^{cre};ROSA$^{mT/mG}$ mice and Csnk1a1$^{fl/fl}$; POMC^{cre};ROSA$^{mT/mG}$ ARCs at E15.5 (A) and P60 (B). (C) Representative micrographs of POMC^{cre};ROSA$^{mT/mG}$ mice and Csnk1a1$^{fl/fl}$;POMC^{cre};ROSA$^{mT/mG}$ PVN at P60.

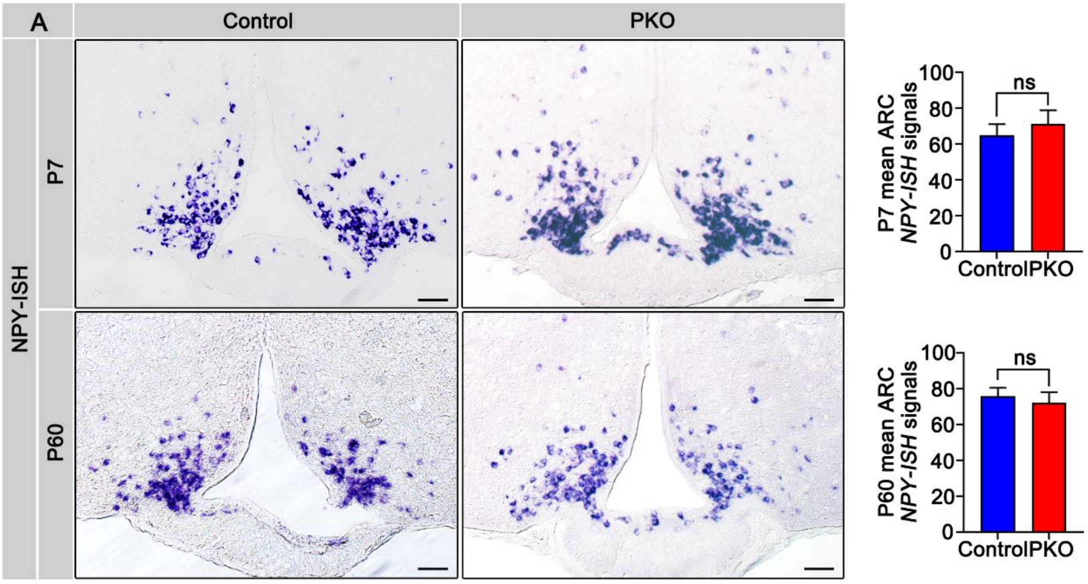

Figure S12. CK1α deletion has no effect on NPY neuron numbers. Related to Figure 4.
(A) NPY-ISH representative micrographs of control and PKO ARCs at P7 and P60 (n = 3, ns P >0.05). Control = Csnk1a1$^{fl/+}$;POMC^{cre}. PKO= Csnk1a1$^{fl/fl}$;POMC^{cre}.

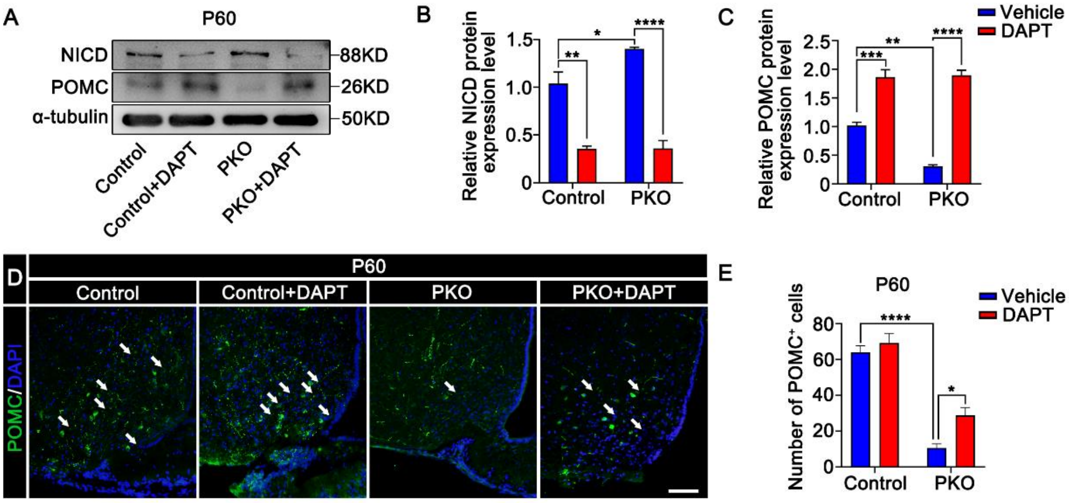

Figure S13. Notch signaling pathway inhibition restores the phenotype of P60 PKO mice. Related to Figure 5.

(A) Western blotting analysis of NICD and POMC protein expression in hypothalami of control and PKO mice i.c.v. injected with aCSF or 0.03 mg/kg DAPT at P60. (B-C) Quantification of NICD (B) and POMC (C) protein in hypothalami at P60 (n = 3, *P < 0.05, **P < 0.01, ***P < 0.001, ****P < 0.0001). (D) POMC immunofluorescence staining in P60 control and PKO hypothalami injected with 0.03 mg/kg DAPT. Scale bars, 50 μm. (E) Numbers of POMC$^+$ neurons in P60 control and PKO hypothalami after injection of aCSF or 0.03 mg/kg DAPT (n = 4, *P < 0.05, ****P < 0.0001). Control = Csnk1a1$^{fl/+}$;POMC^{cre}. PKO = Csnk1a1$^{fl/fl}$;POMC^{cre}.

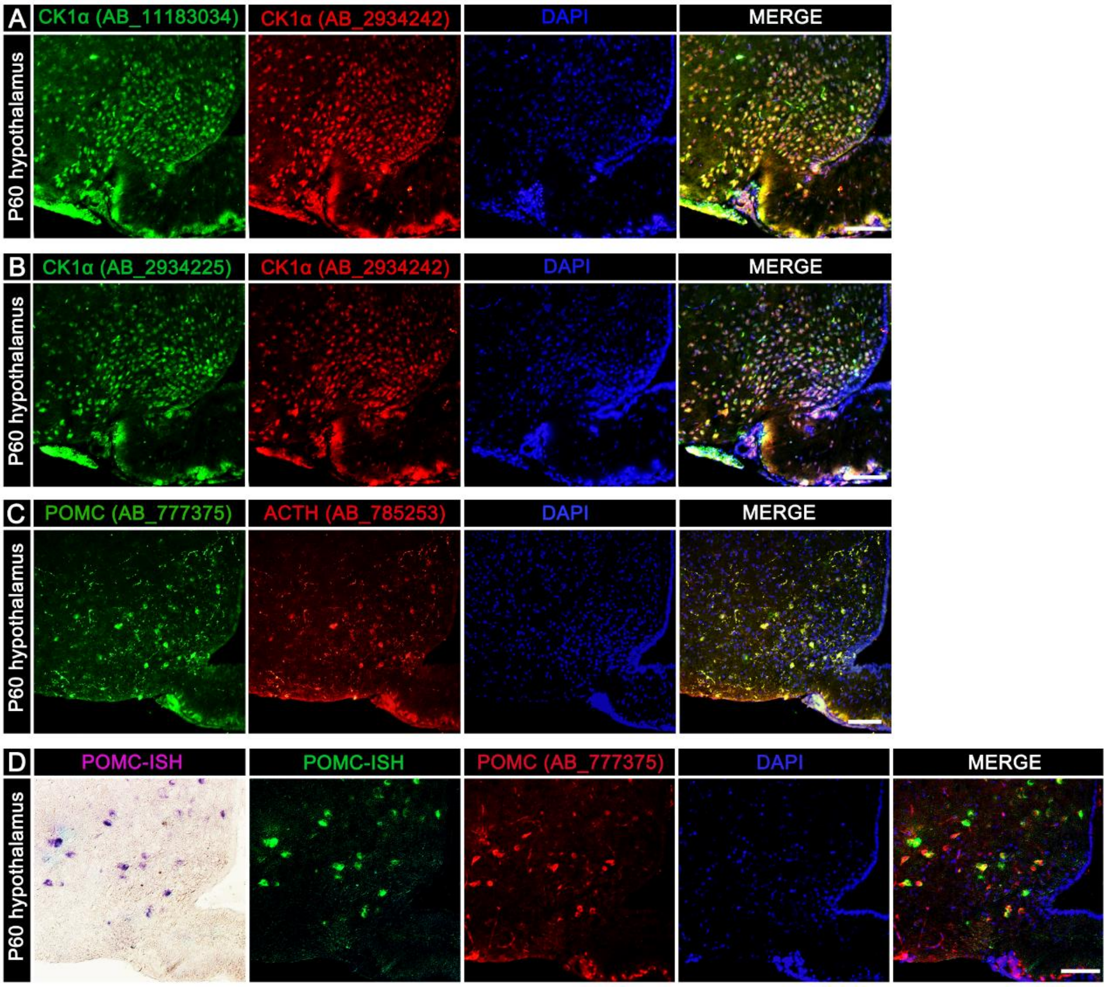

Figure S14. Specificity verification of CK1α and POMC antibodies. Related to STAR Methods.

(A,B) Dual immunofluorescence detection of CK1α (AB_11183034) (green) (A) or CK1α (AB_2934225) (green) (B) with CK1α (AB_2934242) (red) in P60 mouse hypothalamus. (C) Dual immunofluorescence detection of ACTH antibody (AB_785253) (red) and POMC (AB_777375) (green) in P60 mouse hypothalamus. (D) Dual immunofluorescence detection of POMC-ISH (green) and POMC (AB_777375) (red) in P60 mouse hypothalamus. DNA was stained with DAPI. Scale bar, 50 μm.

Table S1. Statistical data of the numbers and percentages of POMC$^+$CK1α$^+$ neurons in E12.5, E15.5 and P60 mouse hypothalamus. Related to Figure 1.

Genotype		Number of POMC$^+$CK1α$^+$ neurons	Number of POMC$^+$ neurons	Percentage
**E12.5**	**Control1**	53	58	93.4%
	**Control2**	61	66	
	**Control3**	70	73	
**E15.5**	**Control1**	74	77	95.6%
	**Control2**	75	79	
	**Control3**	70	73	
**P60**	**Control1**	60	63	96.3%
	**Control2**	65	68	
	**Control3**	57	58	

Table S2. Statistical data of POMC-positive cells in control and PKO mouse hypothalamus at E12.5, E15.5, and P60. Related to Figure 3.

Genotype	Number of POMC$^+$ neurons		
	E12.5	E15.5	P60
Control1	68	67	60
Control2	73	63	72
Control3	74	82	67
PKO1	58	22	13
PKO2	46	18	15
PKO3	54	17	7

Table S3. Statistical data of GFP^{+}POMC^{+} or GFP^{+}POMC^{-} cells in control-ROSA$^{mT/mG}$ and PKO-ROSA$^{mT/mG}$ mouse hypothalamus at E15.5 and P60. Related to Figure 4.

Genotype	E15.5		P60	
	Number of GFP^{+} POMC^{+} neurons	Number of GFP^{+} POMC^{-} neurons	Number of GFP^{+} POMC^{+} neurons	Number of GFP^{+} POMC^{-} neurons
**Control-ROSA$^{mT/mG}$ 1**	43	11	55	12
**Control-ROSA$^{mT/mG}$ 2**	46	22	54	17
**Control-ROSA$^{mT/mG}$ 3**	47	22	44	6
**PKO-ROSA$^{mT/mG}$ 1**	9	70	11	39
**PKO-ROSA$^{mT/mG}$ 2**	12	59	21	43
**PKO-ROSA$^{mT/mG}$ 3**	17	56	13	50

Table S4. Real-time PCR primer sequences. Related to STAR Methods.

Gene	Forward primers (5′-3′)	Reverse primers (5′-3′)
Csnk1a1	CTGGCTCTTTCGGGGACATT	TGCTCTCGTACAGCAACTGG
Gapdh	GGTTGTCTCCTGCGACTTCA	GGGTGGTCCAGGGTTTCTTA
Pomc	CATTAGGCTTGGAGCAGGTC	TCTTGATGATGGCGTTCTTG
Gh	CACGCAAGGGACCAAGTC	TCGGAGCACAGCATTAGAAA
Tshb	AGTAGTGGGTGGAGAAGA	CAGGGAAGGAGAAATAAG
Trh	TCTTGAGGAAAGACCTCCAGCG	AGGCTCCCACTTCTCCCAAATC
Crh	CTCTCTGGATCTCACCTTCC	CTTGTGTGCTAAATGCAGAATC
Mch	GGGGAAAGCCTTTCAGAAG	CTGTGTGGACTCAGCATTC
Hcrt	CTTTCCTTCTACAAAGGTTCCC	GCTTTCCCAGAGTCAGGATAC
PAM	GAATGCCTTGGTACCACCAGACCCATCACTCC	GGAGCCGGGTGGGGGGAGCATTTC
PC1/3	TCCCAGCCCTTCCTACTTGTGT	CTCCGCCGCCCATTCATTAACA
PC2	AAGCCTCCTCCATCAGCCACAT	CCTTGTTCACGCCATCAGCCAT
PRCP	GAACTACCCTTACGCATGCAACT	AATATTGGCACACCTCCTTGATG
Cpe	GCAACGCCCAGGGAATAGAT	GTCTCCTCCGTGCAGATTGG
Bax	AAACTGGTGCTCAAGGCCC	CTTGGATCCAGACAAGCAGC
Bcl2	GAACTGGGGGAGGATTGTGG	GCATGCTGGGGCCATATAGT
Casp3	GAGCTTGGAACGGTACGCTA	GAGTCCACTGACTTGCTCCC
Agrp	GCAAAAGGCATTGAAGAAGC	GGCCTCAAGAAGACAACTGC
Npy	CTCCGCTCTGCGACACTAC	AATCAGTGTCTCAGGGCT
Cart	AAACGCATTCCGATCTACG	GGAAAGAGGGAATATGGGAACC
